# Genome instability triggers intercellular DNA transfer between human cells

**DOI:** 10.1016/j.cell.2026.04.041

**Published:** 2026-05-19

**Authors:** Elizabeth G. Maurais, Alice Mazzagatti, Yu-Fen Lin, Maria Narozna, Qing Hu, Rashmi Dahiya, Derek Santiago-Ferrer, Conor P. Herlihy, Mary Krebs, Nikoleta Pateraki, Evlampia Parcharidou, Stamatis Papathanasiou, Brian J. Beliveau, Gary J. Gorbsky, Isidro Cortés-Ciriano, Peter Ly

**Affiliations:** 1 Children’s Medical Center Research Institute, University of Texas Southwestern Medical Center, Dallas, TX 75390, USA; 2 Department of Pathology, University of Texas Southwestern Medical Center, Dallas, TX 75390, USA; 3 Cell Cycle and Cancer Biology Research Program, Oklahoma Medical Research Foundation, Oklahoma City, OK 73104, USA; 4 Department of Genome Sciences, University of Washington, Seattle, WA 98195, USA; 5 Institute of Molecular Biology, 55128 Mainz, Germany; 6 Brotman Baty Institute for Precision Medicine, Seattle, WA 98195, USA; 7 Institute of Stem Cell and Regenerative Medicine, University of Washington, Seattle, WA 98195, USA; 8 Department of Cell Biology, University of Oklahoma Health Campus, Oklahoma City, OK 73104, USA; 9 European Molecular Biology Laboratory, European Bioinformatics Institute, Hinxton, Cambridge, UK; 10 Somatic Genomics Programme, Wellcome Sanger Institute, Hinxton, Cambridge, UK; 11 Harold C. Simmons Comprehensive Cancer Center, Department of Cell Biology, Department of Pediatrics, University of Texas Southwestern Medical Center, Dallas, TX 75390, USA; 12 Lead contact

## Abstract

The mammalian genome is safeguarded within the confines of the interphase nucleus. However, genomic instability can trigger the mislocalization of nuclear DNA to the cytoplasm within micronuclei or as fragmented chromosomes. Beyond activating cell-autonomous signaling programs, whether such cytoplasmic DNA can elicit non-cell-autonomous consequences to nearby cells remains unclear. Here, we show that cytoplasmic DNAs undergo intercellular transfer through contact-dependent, cytoskeleton-based nanotube structures connecting adjacent human cells. Diverse sources of genomic instability—including exposure to mitotic spindle poisons, ionizing radiation, and Cas9-induced chromosome breakage—promote nanotube-mediated DNA transfer in both cancerous and non-cancerous cells. Transferred DNA fragments are stably inherited as functional extrachromosomal genetic elements in the recipient host genome, thereby conferring heritable phenotypic traits to the recipient cell. Our findings uncover a horizontal gene transfer-like mechanism through which direct cell-cell contact can propagate genomic instability and reshape mammalian genomes.

## In brief

Mammalian cells can pass damaged pieces of their genome directly to neighboring cells via contact-dependent, nanotube-like connections. These transferred DNA fragments persist and function, revealing an unexpected route by which genome damage can propagate between cells.

## Graphical Abstract

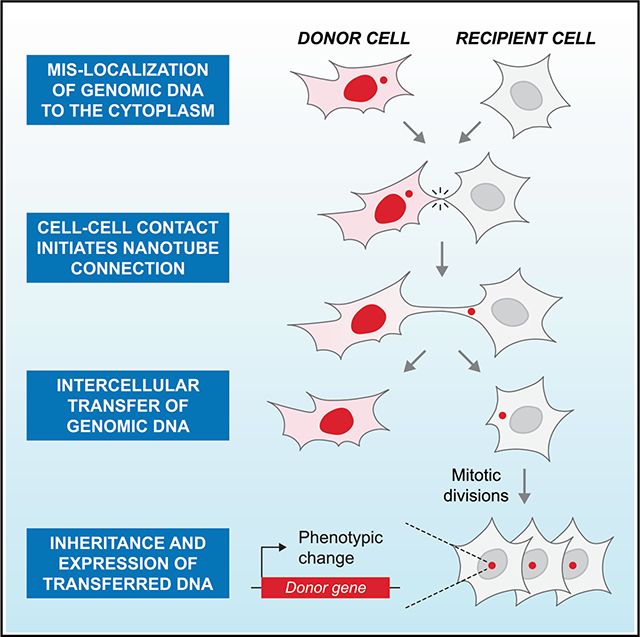

## INTRODUCTION

Mammalian cells can exchange diverse cytoplasmic cargo—including organelles (mitochondria and lysosomes), lipids, ribonucleic acids (mRNAs and microRNAs), and exogenously expressed proteins (GFP and Cre recombinase)—either directly through nanotube connections^1–12^ or indirectly via secreted extracellular vesicles.^13–25^ Nanotube-mediated cargo transfer regulates key physiological processes in eukaryotes, including cardiac morphogenesis and neuronal communication in mice,^26,27^ embryonic development in zebrafish,^28^ and stem cell signaling in flies.^29^ Whether nuclear DNAs are subjected to intercellular transfer via analogous mechanisms has not been documented in a mammalian context. Given the spatial confinement of the genome to the interphase nucleus, we hypothesized that the aberrant mislocalization of nuclear DNA to the cytoplasm may provide a permissive state to enable its transmission between cells.

Genomic instability can cause nuclear DNA to mislocalize to the cytoplasm. For instance, mitotic cell division errors can entrap mis-segregated chromosomes within abnormal cytoplasmic structures called micronuclei. Although micronuclei can trigger p53-dependent cell cycle arrest,^30^ cells that bypass this checkpoint can continue cycling. During interphase, rupture of the micronuclear envelope disrupts proper nucleocytoplasmic compartmentalization and exposes the underlying double-stranded DNA to the cytoplasm.^31^ This can prompt the clearance of micronuclei through autophagy-like mechanisms^32,33^ and/or chromosome breakage via nuclease activity.^34–36^ Micronuclei can also be maintained throughout interphase and persist into the subsequent mitosis,^37^ which triggers an additional burst of DNA damage resulting in catastrophic chromosome fragmentation.^37–41^ This process is termed chromothripsis.^42^

Following mitosis, fragmented chromosomes from micronuclei can reincorporate into the nuclei of one or both daughter cells and undergo error-prone DNA repair to generate genomic rearrangements.^38,40,43,44^ Despite mechanisms that physically tether fragmented chromosomes in proximity during mitosis,^45,46^ some fragments fail to reincorporate into the nucleus and instead mis-accumulate in the cytoplasm.^45,47^ Beyond activation of cell-autonomous signaling programs—such as the cytosolic DNA sensing cyclic GMP-AMP synthase-stimulator of interferon genes (cGAS-STING) pathway^48–50^—it is poorly understood whether cytoplasm-exposed micronuclei and/or cytoplasmic DNA fragments can impact neighboring cells in a non-cell-autonomous manner.

Here, we demonstrate that nuclear DNA mislocalized to the cytoplasm can directly transfer between adjacent human cells through nanotube-like connections, resulting in the intermixing of donor cell DNA with the recipient cell genome. Transferred DNA fragments are genetically stable elements that can be inherited across multiple cell generations and encode functional genes that endow new phenotypic traits to the recipient cell, as shown here by the acquisition of *de novo* drug resistance. Our findings reveal the existence of a horizontal gene transfer-like mechanism in mammalian cells, which could broadly shape our understanding of how cell-cell interactions impact various physiological and diseased states.

## RESULTS

### Intercellular transfer of genomic material from the human cytoplasm

**Figure 1.**
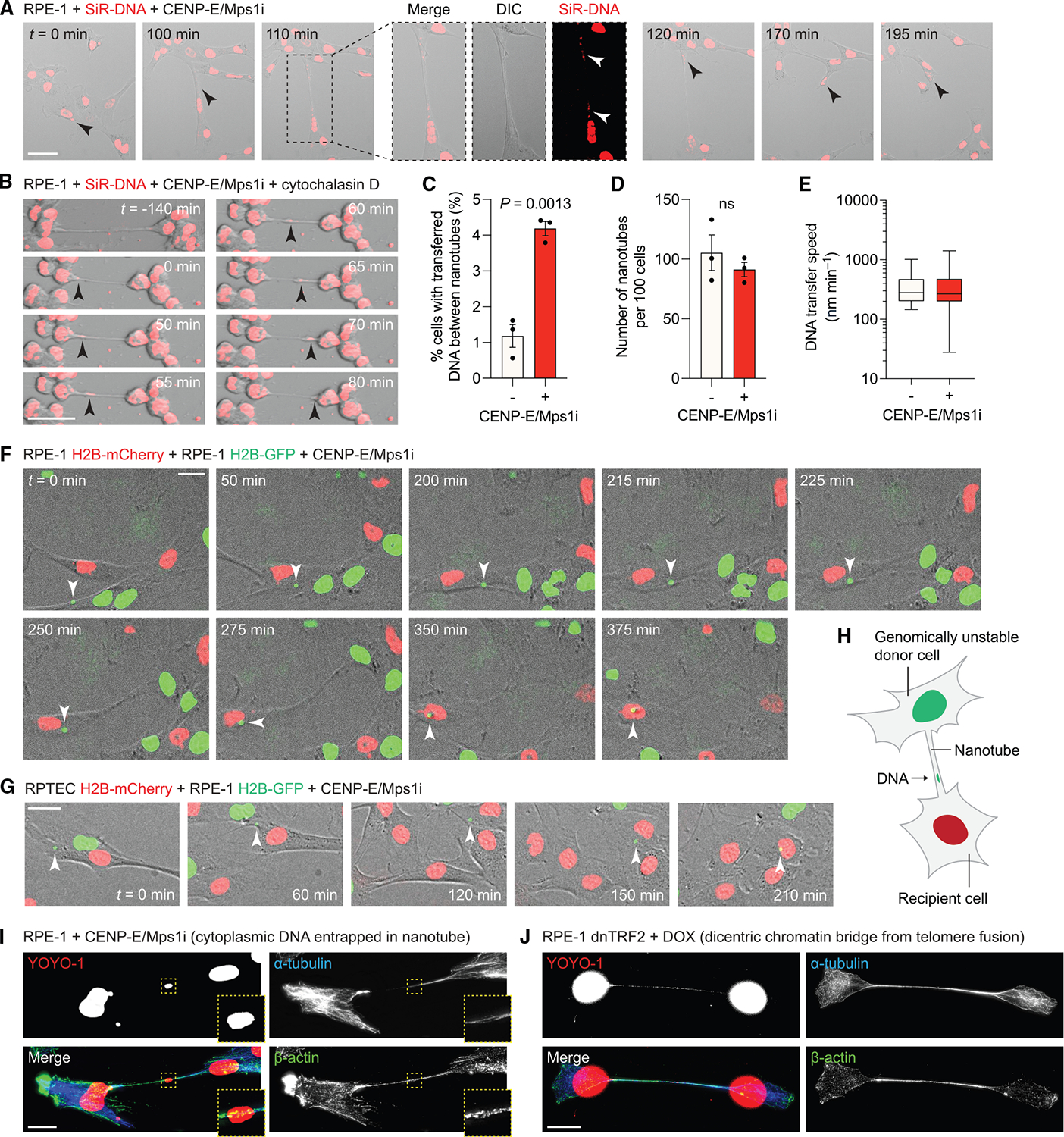
Intercellular DNA transfer via nanotube-like connections in human cells (A) Time-lapse images of RPE-1 cells showing transfer of SiR-DNA-labeled material (arrow) between cells. DIC, differential interference contrast. Scale bars, 50 μm. (B) Time-lapse images of cytochalasin D-treated RPE-1 cells showing transfer of SiR-DNA-labeled material (arrow) between cells. Scale bars, 25 μm. (C) SiR-DNA transfer events detected over a 16-h period in cytochalasin D-treated cells. (D) Nanotubes observed after 120 min of cytochalasin D treatment. For (C) and (D), data represent mean ± SEM from *n* = 3 independent experiments. (E) DNA transfer speed in cytochalasin D-treated cells. Data represent median and 1^st^–99^th^ percentile range from *n* = 13–41 transfer events pooled from 3 independent experiments. (F) Time-lapse images of RPE-1 co-cultures showing transfer of an H2B-GFP-labeled micronucleus (arrow) from an H2B-GFP cell to an H2B-mCherry cell. Scale bars, 20 μm. (G) Time-lapse images of RPE-1 H2B-GFP and RPTEC H2B-mCherry co-cultures showing transfer of an H2B-GFP-labeled micronucleus (arrow) from an RPE-1 cell to an RPTEC. Scale bars, 25 μm. (H) Schematic of nanotube-mediated DNA transfer between a genomically unstable donor cell and a recipient cell. (I) Image of two RPE-1 cells connected by a nanotube containing YOYO-1-stained DNA and immunostained with the indicated cytoskeletal antibodies. Scale bars, 20 μm. (J) Image of two RPE-1 daughter cells connected by an interphase chromosome bridge induced by expression of a dominant-negative TRF2 mutant (dnTRF2) that generates dicentric chromosomes. DOX, doxycycline. Scale bars, 20 μm. For (A)–(G) and (I), cells were co-treated with CENP-E and Mps1 inhibitors (CENP-E/Mps1i) to induce mitotic chromosome segregation errors. See also Figure S1.

To induce mitotic cell division errors, *hTERT*-immortalized, diploid human retinal pigment epithelial (RPE-1) cells were treated with mitotic inhibitors targeting the centromere-associated protein E (CENP-E) motor protein and monopolar spindle 1 (Mps1) kinase, which promotes premature anaphase onset in the presence of misaligned chromosomes.^51^ Surprisingly, using live-cell imaging to monitor cytoplasmic DNA labeled with a double-stranded DNA-specific fluorescent dye (SiR-DNA), we observed the apparent transfer of DNA from the cytoplasm of donor cells to neighboring recipient cells through transient connective structures reminiscent of tunneling nanotubes (Figure 1A; Video S1). These dynamic, tube-like extensions commonly form when two cells come into direct contact and migrate apart.

To enable visualization of DNA transfer events, we prevented cell migration by inhibiting actin polymerization with cytochalasin D. Following treatment with cytochalasin D, RPE-1 cells rapidly constricted and became immobilized, revealing a network of pre-existing nanotubes between adjacent cells that continued to traffic bulky cargo (Figure S1A; Videos S2 and S3). Despite the inability to polymerize actin, immunostaining for α-tubulin showed that most nanotubes remained intact and structurally comprised of microtubules (Figure S1B). Under cytochalasin D-treated conditions, induction of mitotic errors elevated the frequency of detectable SiR-DNA transfer events across nanotubes (Figures 1B and 1C; Video S4). This occurred without affecting the baseline level of spontaneous nanotube formation (Figure 1D), suggesting that nanotube biogenesis is independent of genomic instability. Furthermore, measuring the kinetics of DNA transfer across individual nanotubes—spanning ~10–60 μm in length between adjacent cells—revealed an average transport speed of 390 nm min^−1^ (and 360 nm min^−1^ without mitotic inhibitors) (Figure 1E). These observations motivated further investigation into the possibility that human cells are capable of intercellular DNA transfer.

To avoid toxicity associated with actin inhibition, all further experiments were performed without cytochalasin D. Since cytoplasmic DNAs contain chromatin marks, including phosphorylated histone H2AX,^45^ we expressed fluorescently tagged histone H2B to label and track chromatin by live-cell imaging. To confirm that DNA transfer occurs between two distinct cells, populations of RPE-1 cells labeled with either H2B-GFP or H2B-mCherry were co-cultured at a 1:1 ratio in the presence of mitotic inhibitors and monitored by time-lapse imaging for the transfer of differentially labeled chromatin. Strikingly, in the example shown, an H2B-GFP-labeled micronucleus from an H2B-GFP-expressing RPE-1 cell was directly transferred to an H2B-mCherry-expressing RPE-1 cell, where it remained stably associated with the recipient cell as the donor cell migrated away (Figure 1F; Video S5). To visualize nanotubes with more clearly delineated cell boundaries, we labeled the plasma membrane by expressing HaloTag fused to a CAAX targeting motif (CAAX-Halo) in both H2B-GFP- and H2B-mCherry-labeled RPE-1 cells. Live-cell co-culture imaging revealed similar instances of DNA transfer events traversing through CAAX-Halo-labeled nanotube connections of varying thicknesses^52–54^ (Figure S1C; Videos S6 and S7).

We next determined whether similar transfer events were possible between two distinct epithelial cell lines derived from different tissues. To do so, RPE-1 cells expressing H2B-GFP were co-cultured with *hTERT*-immortalized, diploid human renal proximal tubule epithelial cells expressing a short hairpin RNA against *TP53* (hereafter referred to as RPTECs) and labeled with H2B-mCherry. Indeed, examples of intercellular DNA transfer were observed between differentially labeled RPE-1 cells and RPTECs (Figure 1G; Video S8), demonstrating that cytoplasmic DNA transfer is not restricted to a single cell type and can occur across unrelated epithelial cell types (Figure 1H).

### Genomic instability promotes DNA transfer through nanotube-like connections

Open-ended tunneling nanotubes were initially described in rat pheochromocytoma cells.^1^ Consistent with prior reports,^55,56^ immunofluorescent characterization of non-transformed RPE-1 cells and HeLa cancer cells showed that the mammalian cytoskeleton components α-tubulin and β-actin were a near-universal feature of nanotubes (Figure S1D). Following the induction of mitotic errors, puncta-like DNA signals stained with a sensitive DNA dye (YOYO-1) were readily observed within nanotubes comprised of microtubules and actin in RPE-1 cells (Figure 1I), as well as RPE-1 and HeLa cells expressing fluorescent H2B (Figure S1D). To confirm that DNA-containing nanotubes were distinct from chromatin bridges arising from defective mitoses, we expressed a doxycycline (DOX)-inducible dominant-negative TRF2 (dnTRF2) in RPE-1 cells to uncap telomeres and induce dicentric chromosome fusions.^57^ During mitosis, dicentric chromosomes are pulled in opposing directions by the mitotic spindle and form long chromatin bridges stretched between two daughter cells.^57^ As expected, the puncta-like signals of cytoplasmic DNA trafficking within nanotubes were morphologically distinct from such interphase chromatin bridges whose DNA exhibited an elongated appearance (Figure 1J).

**Figure 2.**
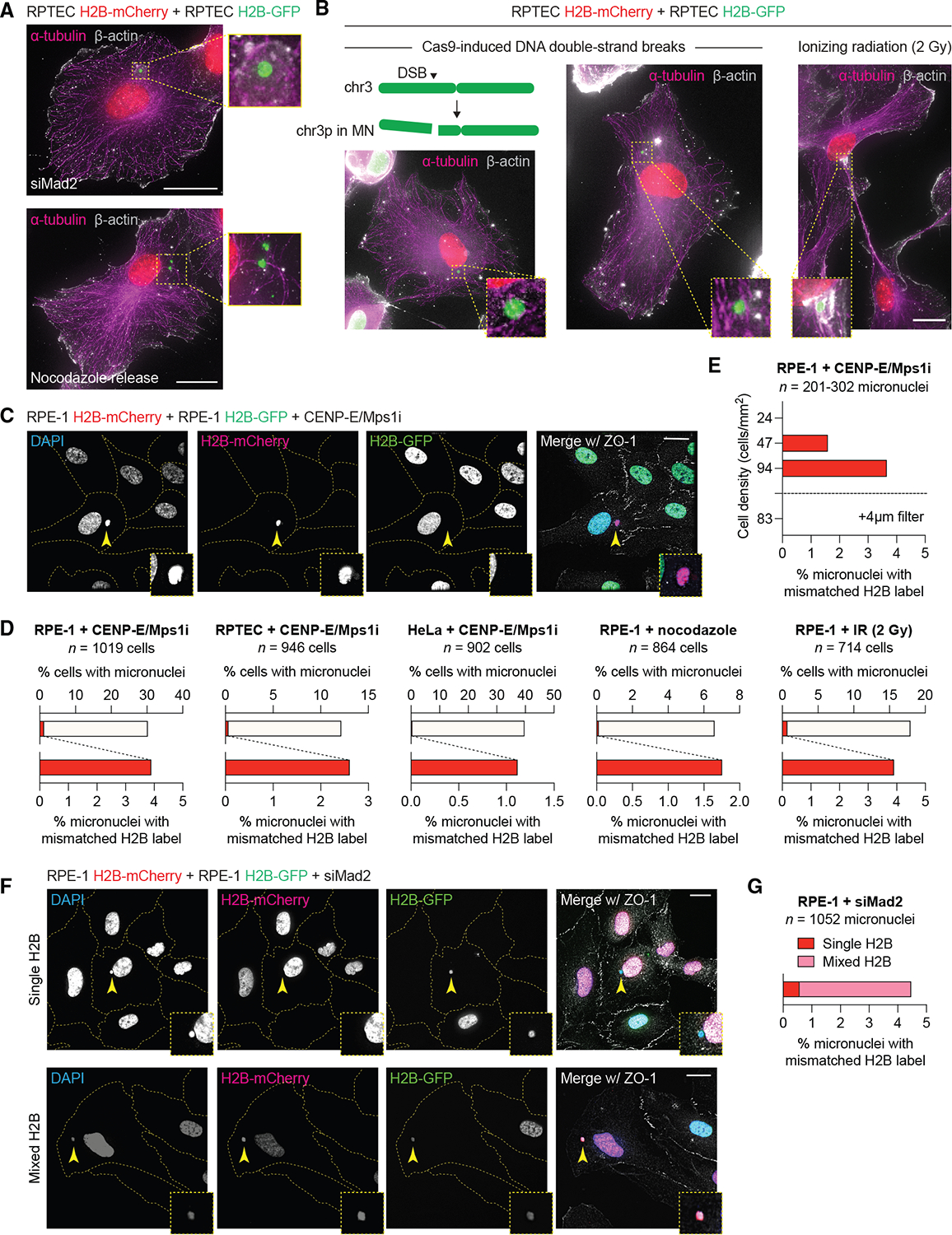
Diverse sources of genomic instability promote intercellular DNA transfer (A) Images of RPTEC co-cultures expressing H2B-mCherry or H2B-GFP containing mismatched H2B-labeled nuclei and micronuclei following depletion of Mad2 (top) or release from nocodazole-induced mitotic arrest (bottom). (B) Images of RPTEC co-cultures expressing H2B-mCherry or H2B-GFP containing mismatched H2B-labeled nuclei and micronuclei following Cas9-induced breakage of chromosome 3p (left and middle) or exposure to ionizing radiation (right). MN, micronuclei. For (A) and (B), cells were immunostained for the indicated cytoskeletal markers. (C) Example of mismatched H2B labeling in RPE-1 H2B-mCherry and RPE-1 H2B-GFP co-cultures treated with mitotic inhibitors and immunostained for ZO-1 to label cell boundaries. (D) Total and mismatched micronuclei from the indicated conditions; data from the indicated number of cells pooled from 3 independent experiments. (E) Mismatched micronuclei at the indicated cell densities with or without separation of the co-cultured populations using a transwell filter; data from *n* = 201–302 micronuclei pooled from 3 independent experiments. (F) Examples of mismatched H2B labeling in RPE-1 H2B-mCherry and RPE-1 H2B-GFP co-cultures depleted of Mad2 and immunostained for ZO-1 to label cell boundaries. Top panel shows an H2B-mCherry-labeled cell harboring a mismatched H2B-GFP-labeled micronucleus (arrow). Bottom panel shows an H2B-mCherry-labeled cell harboring a mixed mCherry-/GFP-labeled micronucleus (arrow). (G) Quantification of (F); data from the indicated number of micronuclei pooled from 3 independent experiments. All scale bars, 20 μm. See also Figure S2.

Live-cell imaging of co-cultures between cells expressing differentially labeled chromatin markers suggested that successful DNA transfer events would yield recipient cells exhibiting mismatched labeling of H2B signals between the nucleus and DNA in the cytoplasm (Figures 1F–1H; Videos S5, S6, S7, and S8). In agreement, mismatched-labeled H2B signals were detectable across multiple cell lines (RPE-1, RPTEC, and HeLa) treated with mitotic inhibitors (Figure S1E). To determine whether alternative sources of mitotic perturbations can instigate intercellular DNA transfer, we accelerated mitotic progression by transiently depleting the spindle assembly checkpoint protein Mad2 or inducing prolonged mitotic arrest (up to 6 h) using the microtubule polymerization inhibitor nocodazole, which generates improper spindle-kinetochore attachments upon microtubule repolymerization. Both Mad2-depleted and nocodazole-released RPTECs exhibited chromatin puncta traversing through nanotubes (Figures S2A and S2B), which further generated mismatched-labeled H2B signals between the nucleus and cytoplasm of the recipient cell (Figure 2A). This is consistent with mitotic errors as a general source of cytoplasmic DNAs that are subjected to intercellular transfer via nanotubes in both non-transformed and cancer cells.

To test whether interphase DNA damage represents an additional source of genomic instability-mediated DNA transfer, we used CRISPR-Cas9 to generate a site-specific DNA double-strand break (DSB) on chromosome 3p, producing an acentric chromosome arm that mis-segregates into micronuclei when left unrepaired into mitosis.^58^ In the example shown in Figure S2C, a micronucleated mother RPTEC labeled with H2B-mCherry is divided into two daughter cells (each marked by yellow asterisks). The micronucleus persisted in one of the daughters, which then transferred to a nearby cell (marked by a magenta asterisk), indicative of the transfer of H2B-labeled chromatin as an on-target consequence of CRISPR editing. This was further confirmed in fixed co-cultures expressing distinct H2B reporters following the induction of Cas9-induced DSBs on chromosome 3p, which provoked the entrapment of DNA in nanotubes (Figure S2D) and a corresponding mismatch in H2B labeling between the nucleus and cytoplasm (Figure 2B). To extend this to another source of DNA damage, we exposed RPTEC co-cultures to low-dose (2 Gy) ionizing radiation (IR) to generate random DSBs throughout the genome. Indeed, DNA damage induced by IR similarly promoted mismatched H2B signals (Figures 2B and S2E), consistent with mitotic perturbations (Figure 2A) and Cas9-induced DSBs (Figure 2B). Thus, genomic instability from DNA damage generates cytoplasmic DNAs that are susceptible to nanotube-mediated intercellular DNA transfer.

We next quantified the frequency of detectable DNA transfer events in co-cultures of H2B-mCherry- and H2B-GFP-labeled cell lines following the induction of mitotic errors. Immunostaining for the tight junction protein Zonula Occludens-1 (ZO-1) enabled demarcation of the outer cell membrane and examination for mismatched H2B-labeled nuclei and micronuclei. Inspection of 2,867 RPE-1, RPTEC, and HeLa cells treated with mitotic inhibitors (pooled from 3 independent experiments) revealed that 1.1%–3.9% of micronuclei harbored mismatched-labeled cytoplasmic H2B signals compared with their corresponding nucleus (Figures 2C and 2D). These measurements are underestimated (by half) as DNA transfer between cells expressing the same H2B reporter is undetected. Other sources of genomic instability resulting in micronuclei, such as release from nocodazole-induced mitotic arrest or IR exposure, triggered mismatched H2B signals within a similar frequency range in RPE-1 cells (Figure 2D).

Notably, DNA transfer frequency declined in a manner corresponding to decreased cell density, consistent with direct cell-cell contact as a requirement for initial nanotube formation (Figure 2E). In agreement, the inclusion of a 4-μm pore transwell filter to physically separate the H2B-mCherry- and H2B-GFP-labeled RPE-1 populations abolished detectable DNA transfer events (Figure 2E). A proportion of transferred micronuclei (26.7%) were encapsulated by the nuclear membrane component Lamin B1 (Figure S2F), indicating that both lamina-coated and uncoated cytoplasmic DNAs are capable of transfer. Furthermore, to determine whether donor histones are replaced by recipient histones during DNA replication, we examined mismatched cytoplasmic H2B signals in longer-term co-cultures following 3-day depletion of Mad2. Indeed, the majority (87.2%) of transferred micronuclei acquired mixed mCherry- and GFP-labeled H2B signals over time (Figures 2F and 2G), suggestive of recipient histone incorporation during DNA synthesis.

### Intercellular DNA transfer occurs across cell types and chromosomes

**Figure 3.**
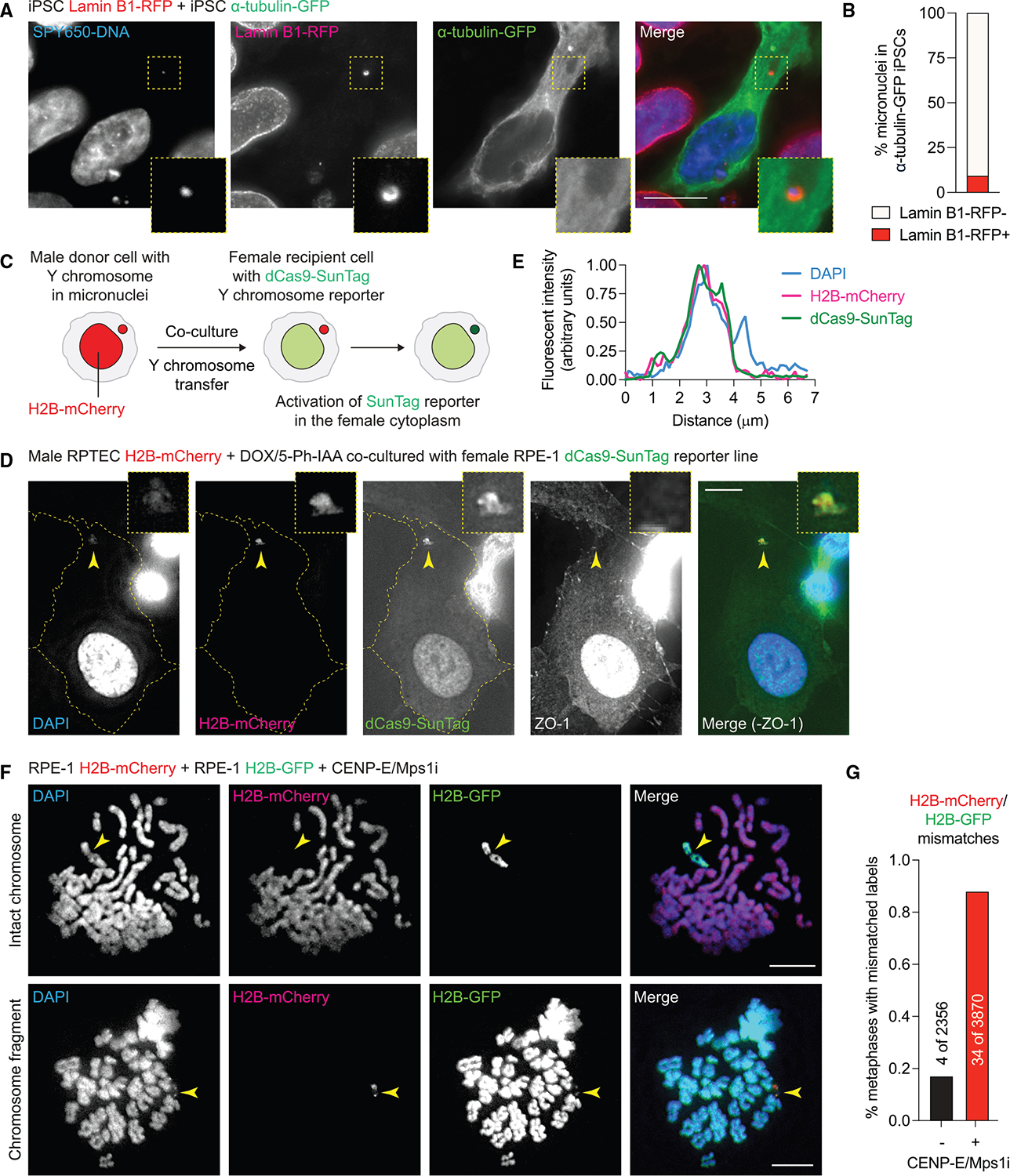
Transferred DNA enters the recipient cytoplasm and intermixes with host chromosomes during mitosis (A) Example of Lamin B1-RFP iPSCs co-cultured with α-tubulin-GFP iPSCs. Magnified inset depicts a Lamin B1-RFP-labeled micronucleus within the cytoplasm of an iPSC expressing α-tubulin-GFP. (B) Quantification of (A); data from *n* = 294 micronucleated α-tubulin-GFP iPSCs pooled from 3 independent experiments. (C) Experimental schematic to detect the transfer of Y chromosome-containing sequences from male donor cells to female recipient cells expressing a dCas9-SunTag reporter targeting the DYZ1 repetitive array of the Y chromosome. (D) Example of a DAPI- and H2B-mCherry-labeled micronucleus recognized by the dCas9-SunTag reporter within the cytoplasm of the female RPE-1 cell reporter line. Y chromosome micronuclei were induced by inactivating the Y centromere using a CENP-A replacement strategy (detailed in Figure S3). (E) Line scan analysis of fluorescence intensity through the arrow in (D). (F) Images of metaphase spreads from RPE-1 co-cultures showing an H2B-mCherry-labeled metaphase containing an H2B-GFP-labeled chromosome (top) or an H2B-GFP-labeled metaphase containing mixed mCherry-/GFP-labeled chromosome fragments (bottom). (G) Quantification of (F); data from the indicated number of metaphase spreads pooled from 3 independent experiments. All scale bars, 10 μm. See also Figures S3 and S4.

We next asked whether intercellular DNA transfer extends beyond somatic epithelial cells. To address this, we performed complementary experiments in human induced pluripotent stem cells (iPSCs), which harbor a low (~2%–3%) baseline rate of micronucleation.^59^ iPSCs expressing an endogenously tagged allele of the nuclear membrane marker Lamin B1 fused to mTagRFP (Lamin B1-RFP) were co-cultured with iPSCs expressing endogenous α-tubulin fused to mEGFP (α-tubulin-GFP). We then examined α-tubulin-GFP iPSCs as recipients for cytoplasmic DNA labeled with Lamin B1-RFP. Among 294 micronucleated α-tubulin-GFP iPSCs (pooled from 3 independent experiments), 9.2% of micronuclei harbored Lamin B1-RFP (Figures 3A and 3B), consistent with its transfer from a Lamin B1-RFP-expressing donor cell. This frequency is likely underestimated, as only half (46.9%) of micronuclei in Lamin B1-RFP iPSCs incorporate detectable Lamin B1. The higher transfer frequency compared with somatic epithelial cells may reflect the use of Rho-associated protein kinase (ROCK) inhibitors during routine iPSC culture, which enhances nanotube formation.^9^ Thus, intercellular DNA transfer occurs spontaneously in human iPSCs in unperturbed conditions.

To examine whether intercellular DNA transfer can be induced on a specific target chromosome, we engineered a previously established centromere-selective inactivation strategy targeting the human Y chromosome for mis-segregation^39^ in male RPTECs. To do so, we first expressed the centromeric histone variant CENP-A fused to GFP and a mini-auxin-inducible degron (mAID-GFP-CENP-A) and used CRISPR-Cas9 editing to inactivate both endogenous *CENPA* alleles (*CENPA*^−*/*−^). mAID-GFP-CENP-A was rapidly degraded within 30 min following induction with the synthetic auxin analog 5-Ph-IAA (Figures S3A and S3B). Next, we introduced a DOX-inducible CENP-A carboxy-terminal tail mutant that prevents kinetochore assembly on the Y centromere, thereby triggering its inactivation^39^ (Figure S3C). Multiplex DNA fluorescence *in situ* hybridization (FISH) confirmed that these genetically modified RPTECs remained genomically stable and diploid (Figure S3D). As expected, treatment with DOX/5-Ph-IAA induced Y chromosome mis-segregation into micronuclei with high efficiency and specificity, as determined by DNA FISH (Figures S3E and S3F). Co-culturing of H2B-mCherry- and H2B-GFP-labeled RPTECs demonstrated that Y chromosome-containing micronuclei can indeed traffic via nanotubes (Figure S3G) and generate cells harboring mismatched-labeled H2B signals (Figure S3H). Altogether, diverse sources of genomic instability—including mitotic checkpoint inactivation, prolonged mitotic arrest, Cas9-induced DNA damage, IR exposure, and centromere inactivation—promote intercellular DNA transfer across different human cell types.

### Transferred DNA enters the recipient cytoplasm and intermixes with host chromosomes during mitosis

To confirm that transferred DNAs are deposited into the cytoplasm of recipient cells, we engineered female RPE-1 cells to express a Y chromosome-specific reporter. This system is comprised of a nuclease-deficient dCas9 fused to a SunTag (dCas9-SunTag), which targets and labels the DYZ1 repetitive array (spanning 2,000 to 4,000 repeats) on the long arm of the Y chromosome with multiple GFP molecules.^45,60^ Female RPE-1 dCas9-SunTag reporter cells were then co-cultured with male RPTECs expressing H2B-mCherry and containing Y chromosome-specific micronuclei induced by centromere inactivation, as described above (Figure S3). Induction of Y chromosome mis-segregation produced robust dCas9-SunTag-labeled puncta that colocalized with DAPI and H2B-mCherry signals (Figures 3D and 3E), indicating the successful transfer of Y chromosome-derived sequences from donor RPTECs into the cytoplasm of female recipient RPE-1 reporter cells.

We predicted that transferred DNA in the recipient cytoplasm might incorporate into the host genome upon nuclear envelope breakdown during subsequent mitosis. To test this, H2B-mCherry and H2B-GFP RPE-1 co-cultures were treated with mitotic inhibitors, and metaphase chromosome spreads were analyzed for mismatched H2B labeling one cell cycle later. Figure 3F shows an example of an H2B-GFP-labeled mitotic chromosome containing an apparent centromere (marked by a visible primary constriction, as indicated by the arrow) within an H2B-mCherry-labeled metaphase spread (top panel), as well as an example of H2B-mCherry-labeled chromatin fragments within a singularly labeled H2B-GFP recipient cell genome (bottom panel). Such mismatches were detected at an increased frequency in the presence of mitotic inhibitors (0.88%, *n* = 3,870 metaphase spreads pooled from 3 independent experiments) compared with controls (Figure 3G).

To more closely examine the behavior of transferred DNA during mitosis, we transiently synchronized RPE-1 co-cultures in early mitosis with nocodazole and allowed them to release into metaphase. Immunostaining of mitotic spindle microtubules revealed a similar frequency of individual chromosomes or chromosome fragments harboring mismatched H2B labeling (1.3%, *n* = 873 mitotic cells), including those that were improperly aligned during metaphase and anaphase (Figure S4A). To determine whether such mitotic misalignment defects were caused by the transfer of acentric fragments, we examined co-cultures of H2B-mCherry RPTECs with H2B-GFP RPE-1 cells (Figure S4B) and H2B-GFP and H2B-mCherry RPE-1 cells (Figure S4C) for the centromeric marker CENP-A. Indeed, whereas some mismatched H2B signals in RPE-1 co-cultures contained a detectable centromere signal (3 of 11), most lacked CENP-A (8 of 11)—consistent with the transfer of both centromere-containing and acentric fragments.

As a complementary strategy to labeling chromatin, we next used differential labeling with thymidine nucleotide analogs to directly visualize mismatched DNA on metaphase chromosome spreads (Figure S4D). RPE-1 cells were pre-labeled with either iododeoxyuridine (IdU) or chlorodeoxyuridine (CldU) for three cell cycles, which successfully incorporated into most individual chromatids (Figure S4E). Following co-culture of IdU- and CldU-labeled RPE-1 cells treated with mitotic inhibitors, inspection of 759 metaphase spreads (pooled from 3 independent experiments) revealed mismatched DNA fragments in 1.7% of mitotic cells (Figures S4F and S4G). These data show that transferred DNA can intermix with recipient cell chromosomes during the subsequent mitosis.

### Transferred DNA fragments form heritable and functional extrachromosomal elements in recipient cells

**Figure 4.**
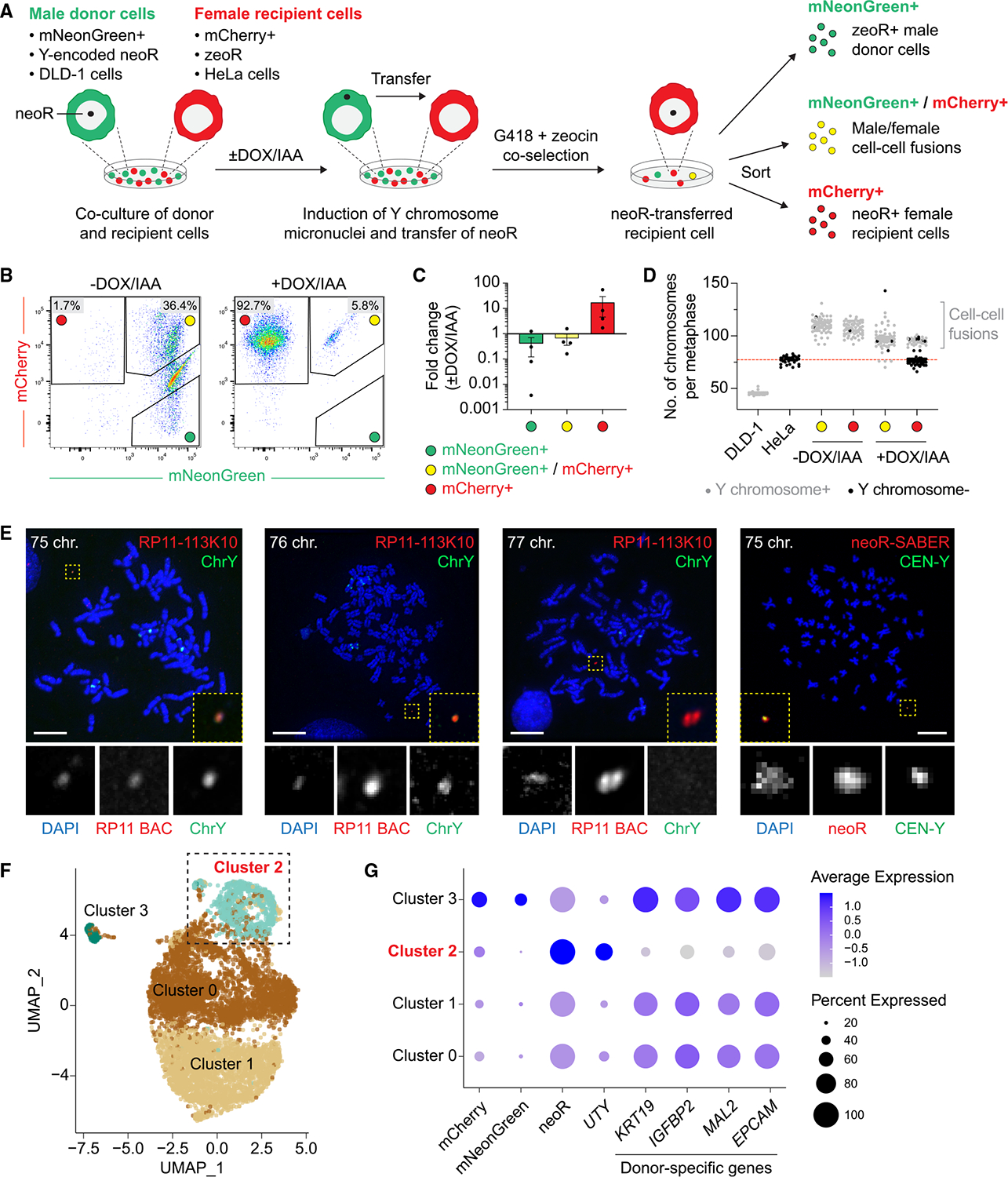
Transferred DNA fragments confer heritable phenotypic traits to recipient cells (A) Experimental schematic to isolate female recipient cells following intercellular transfer of a Y chromosome-encoded antibiotic resistance gene (neoR) induced by centromere inactivation. Male donor and female recipient cells expressing unique fluorescent and selection markers were co-cultured, co-selected, and sorted as indicated. (B) Representative flow cytometry scatter plots from the co-cultures outlined in (A) treated with or without DOX/IAA. See Figure S5A for the gating strategy using parental donor and recipient cells. (C) Fold change in the indicated cell populations in (B) relative to untreated controls. Data represent mean ± SEM of *n* = 4 independent experiments. (D) Total number of chromosomes per metaphase spread from FACS-isolated populations with or without an intact Y chromosome. The red dashed line indicates the mean number of chromosomes in the parental recipient cell line. Data from *n* = 29–103 metaphases per condition. (E) Representative metaphase spreads prepared from DOX/IAA-treated, G418/zeocin co-selected cells following FACS isolation. DNA FISH detects a single-copy, extrachromosomal Y chromosome-derived fragment hybridizing to the indicated probes targeting the Yq11.221 locus encoding neoR. The total number of chromosomes per metaphase spread is indicated. The green chromosome paint signals observed on chromosomes reflect cross-hybridization to homologous sequences on the X chromosome. BAC, bacterial artificial chromosome; SABER, signal amplification by exchange reaction. Scale bars, 10 μm. (F) UMAP embedding of single-cell transcriptomes (*n* = 7,601 cells) from the isolated mCherry+ population shown in (A). Each point represents a single cell labeled by cluster assignments inferred through unsupervised cell clustering (cluster 0: 3,676 cells; cluster 1: 2,919 cells; cluster 2: 902 cells; and cluster 3: 104 cells). (G) Dot plot summarizing the average expression of mCherry, mNeonGreen, neoR, *UTY*, and the indicated donor-specific autosomal genes with undetectable mRNA levels in the parental recipient cells. Each row represents a cluster from (F). Cluster 2 corresponds to mCherry+ cells with detectable neoR and *UTY* transcripts but lacking expression of donor-specific autosomal genes. See also Figures S5 and S6

It remains unclear whether transferred DNAs were heritable and functional in the long term. However, we reasoned that the transfer, maintenance, and expression of an antibiotic selection marker may enable enrichment for rare yet stable DNA transfer events in a subpopulation of recipient cells. To test this, we designed an experimental strategy (Figure 4A) leveraging previously engineered male DLD-1 colorectal cancer cells whose Y chromosome centromere can be inactivated by CENP-A replacement^39^ as a donor cell. Importantly, the Yq11.221 locus encodes a CRISPR-engineered neomycin-resistance (neoR) gene conferring resistance to the antibiotic G418.^43^ These male donor cells were further modified to express mNeonGreen and co-cultured with G418-sensitive female HeLa recipient cells expressing mCherry and a zeoR gene conferring resistance to zeocin (Figure S5A). Y chromosome mis-segregation into micronuclei was induced in the male donor cells by the addition of DOX/IAA to the co-culture medium for three days. neoR transfer events were subsequently enriched by co-selection with G418 and zeocin for up to four weeks, followed by expansion of resistant cells. This procedure yielded three potential populations that can be separated by fluorescence-activated cell sorting (FACS), as illustrated in Figure 4A (see also STAR Methods). Under these conditions, successful transfer of the Y-encoded neoR gene from parental male donors to female recipients is expected to generate mCherry-labeled cells that acquired *de novo* resistance to G418.

Flow cytometry revealed three distinct populations (Figure 4B), including an mCherry+/mNeonGreen+ population indicative of spontaneous cell-cell fusion. Whereas control conditions yielded predominantly cell-cell fusion events due to co-selection, induction of Y chromosome mis-segregation with DOX/IAA increased the population of mCherry+ cells by up to 55-fold compared with controls (Figures 4B and 4C). As an internal control, mNeonGreen+ male cells that became resistant to zeocin owing to spontaneous acquisition of zeoR were not enriched (Figures 4B and 4C). Analyses of total chromosome number seven days post-FACS showed that the expected cell-cell fusions exhibited a karyotype approaching the cumulative number of chromosomes from both male donor and female recipient cells (Figure 4D). By contrast, the mCherry+ population harbored two distinct subpopulations: one harbored a lower chromosome count that is consistent with the initial karyotype of the parental female recipient line, and another exhibited a cell-cell fusion karyotype (Figure 4D). Metaphase FISH using chromosome-specific paint probes confirmed that the expected cell-cell fusion subpopulation possessed an intact copy of the Y chromosome (gray dots) (Figure 4D).

To confirm the presence of Y chromosome-derived DNA within the mCherry+ population, we designed PCR primers targeting the neoR cassette and adjacent sequences,^43^ which detected the CRISPR-edited Yq11.221 locus in bulk genomic DNA (Figures S5B and S5C). To identify the physical genomic location of the neoR locus, we performed cytogenetic analyses using (1) a bacterial artificial chromosome (BAC) probe targeting the Yq11.221 locus and Y chromosome-specific paint probes and (2) synthesized oligonucleotide probes directly spanning the neoR cassette along with ~15 kilobases of flanking Yq11.221 sequences and amplified by signal amplification by exchange reaction (SABER).^61^ The specificity of the latter neoR-SABER probes in combination with a Y centromere-specific probe (located ~3 megabases apart) was validated in the male donor cells (Figure S5D). Inspection of metaphase spreads derived from mCherry+ cells revealed the presence of single-copy, extrachromosomal DNA (ecDNA) fragments derived from the targeted Yq11.221 locus that hybridized to the BAC and SABER probes in 8/240 (3.3%) and 7/103 (6.8%) metaphases, respectively (Figure 4E). These frequencies likely represent a lower bound, given the sensitivity limitations of microscopy-based approaches in detecting small and heterogeneous DNA fragments. Notably, Yq11.221-derived ecDNA was detected exclusively in cells whose total chromosome number closely matched that of the parental female recipient cells (Figure 4D), consistent with intercellular DNA transfer without cell-cell fusion.

Having established the presence of transferred Yq11.221-derived DNA fragments in recipient cells, we asked whether these fragments were transcriptionally functional. We examined the gene products of neoR and *UTY*—a somatically expressed Y-chromosome gene located ~200 kilobases from the neoR cassette (Figure S5B). Reverse transcription PCR (RT-PCR) and immunoblotting detected both mRNA transcripts and protein, respectively, encoded by neoR and *UTY* in bulk mCherry+ cells but not in the corresponding controls (Figures S5E and S5F). Lastly, to assess transcriptional activity at single-cell resolution, we performed single-cell RNA sequencing (scRNA-seq) on parental donor and recipient cells compared with the isolated mCherry+ population. Unsupervised clustering and UMAP (uniform manifold approximation and projection) analysis showed clear segregation of parental donor and recipient transcriptomes, including donor-enriched autosomal genes (e.g., *KRT19*, *IGFBP2*, *MAL2*, and *EPCAM*) that distinguished the two parental lines (Figure S6A). As expected for exogenous reporters expressed at modest levels, mCherry and mNeonGreen transcripts were detected in only a subset of the corresponding parental cells, consistent with incomplete transcript capture and stochastic gene dropout intrinsic to scRNA-seq for low-abundance transcripts.

Importantly, within the mCherry+ population, scRNA-seq revealed a distinct subpopulation (cluster 2, 11.9% of cells; Figure 4F) that contained transcripts derived from the Yq11.221 locus, including neoR and *UTY*, while lacking robust expression of donor-enriched genes (Figures 4G and S6B). Although some donor-associated transcripts were detectable in a minor fraction of cluster 2 cells, their expression levels were substantially lower than in the donor population. In contrast, the remaining clusters (clusters 0, 1, and 3) co-expressed both donor- and recipient-specific genes at high levels, consistent with the cell-cell fusion subpopulation previously observed by cytogenetics (Figure 4D). Altogether, these results demonstrate that transferred DNA sequences are maintained throughout multiple cell generations and support functional gene expression, in turn conferring new phenotypic traits.

## DISCUSSION

Horizontal gene transfer is well established in bacteria,^62–66^ archaea,^67,68^ and, to a lesser extent, in plants^69^ and insects.^70^ Although direct evidence in a human context remains limited, prior studies have implicated that mammalian cells can uptake telomeric sequences, plasmid DNA, and nuclear bud-like structures that are secreted into the extracellular environment.^71–75^ Additionally, intercellular mitochondrial transfer inherently entails the co-transfer of mitochondrial DNA, which can impact recipient cell metabolism.^3,4,7,9,10,18,19,21,22,76–78^ Here we show that nuclear DNA transfer occurs at low yet measurable frequencies across human somatic cell lines and iPSCs, irrespective of the source of genomic instability, ranging from whole-chromosome segregation errors during mitosis to the induction of genomewide DNA damage. In tumors, this could arise intrinsically from chromosomal instability and replication stress or extrinsically via exposure to anti-mitotic agents, radiotherapy, and chemotherapy. In a manner analogous to bacterial conjugation, the transfer of functional genetic elements through physical cellular connections can confer new phenotypic traits, as exemplified here by the acquisition of drug resistance in otherwise drug-naive recipient cells.

Intercellular DNA transfer may have a wide-ranging impact on how cell-cell interactions shape mammalian genomes. In principle, such events could serve as a previously unrecognized source of somatic aneuploidy and DNA copy number alterations, including chromosome loss or segmental deletions in donor cells. Conversely, DNA transfer could generate genomic alterations in recipient cells that resemble whole-chromosome gains or non-tandem duplications, challenging the assumption that these changes originate exclusively from cell-autonomous mitotic defects and DNA replication errors, respectively. Transferred DNA fragments that circularize can also become propagated as ecDNA elements that commonly amplify oncogenes or genes conferring drug resistance. Lastly, the tumor microenvironment comprises a complex ecosystem of unique cell types. This mechanism could potentially enable genomically unstable cancer cells to disseminate oncogenic alleles, deleterious mutations, and/or regulatory elements to neighboring non-cancerous cells. We anticipate that such an exchange of genetic material may have a profound impact on human biology.

Many questions regarding the mechanisms and consequences of this process remain outstanding. For example, why do human cells establish nanotube connections, and is DNA movement within these structures a stochastic or active, motor-driven process? Following DNA transfer, it is unclear whether cytoplasmic DNA received by recipient cells engages innate immune sensors such as the cGAS-STING signaling pathway. In the context of cancer, how DNA transfer shapes clonal heterogeneity and the mutational landscape of somatic genomes remains unknown. Detectable transfer events occur at low frequency in cultured cells yet may still exert meaningful biological consequences under selection, and the extent to which this contributes to genome evolution or disease progression *in vivo* remains undetermined. Together, these observations highlight the broader implications of intercellular DNA transfer for mammalian evolution, physiology, and disease.

### Limitations of the study

Our findings demonstrate that nanotube-mediated intercellular DNA transfer requires direct cell-cell contact; however, we cannot fully exclude alternative contact-independent mechanisms—such as extracellular vesicles—that might transport DNA fragments below the detection limits of the approaches used here. Additionally, the molecular determinants of nanotube biogenesis, cargo selection, and transport dynamics remain undefined, and a consensus framework for classifying functionally distinct nanotube subtypes has yet to be established. Consistent with this, our attempts to genetically and pharmacologically inhibit nanotube formation were unsuccessful, underscoring the need for a deeper understanding of nanotube biology.

## RESOURCE AVAILABILITY

### Lead contact

Further information and requests for resources and reagents should be directed to and will be fulfilled by the lead contact, Peter Ly (peter.ly@utsouthwestern.edu).

### Materials availability

Cell lines, plasmids, and SABER-FISH probe sequences generated in this study are available upon request.

### Data and code availability

- All data reported in this paper will be shared by the lead contact upon request.
- Single-cell RNA sequencing data have been deposited in the European Nucleotide Archive (ENA) at EMBL-EBI under accession number PRJEB107007.
- This paper does not report original code.
- Any additional information required to reanalyze the data reported in this paper is available from the lead contact upon request.

## STAR★METHODS

### EXPERIMENTAL MODEL AND STUDY PARTICIPANT DETAILS

All cell lines were maintained at 37 °C in a humidified incubator with 5% CO_2_ and atmospheric oxygen. DLD-1, HeLa S3, HEK293GP, and HEK293T cells were cultured in Dulbecco’s Modified Eagle Medium (DMEM; Gibco) supplemented with 10% tetracycline-free fetal bovine serum (FBS; Omega scientific) and 100 U mL^−1^ penicillin–streptomycin. RPE-1 cells were maintained in DMEM/F-12 (Gibco) supplemented with 10% FBS and 100 U mL^−1^ penicillin–streptomycin. RPE-1 dnTRF2 cells were a gift from John Maciejowski. Renal proximal tubule epithelial cells (RPTECs, a gift from Denise Marciano) expressing an shRNA against *TP53* were cultured in Renal Epithelial Cell Growth Basal Medium (Lonza) supplemented with 2.5% tetracycline-free FBS, 100 U mL^−1^ penicillin–streptomycin, 1x insulin–transferrin–selenium (Gibco), 10 ng mL^−1^ human recombinant epidermal growth factor, 1 μg mL^−1^ hydrocor- tisone, 50 ng mL^−1^ triiodo-L-thyronine, 30 μg mL^−1^ gentamicin, and 15 ng mL^−1^ amphotericin B. All cell lines were authenticated by karyotyping and routinely tested for mycoplasma contamination using the Universal Mycoplasma Detection Kit (ATCC).

The following human induced pluripotent stem cell (iPSC) lines derived from the WTC-11 parental line at the Allen Institute for Cell Science were used in this study: AICS-0012 cl. 105 (TUBA1B–mEGFP; Cat. #AICS-0012; Coriell Institute for Medical Research, RRID: CVCL_IR34) and AICS-0034 cl. 62 (LMNB1–mTagRFP-T; Cat. #AICS-0034–062; Coriell Institute for Medical Research, RRID: CVCL_ZX21). Cell lines were obtained through the Allen Cell Collection (Coriell Institute for Medical Research). iPSCs were maintained in 12.5 cm^2^ flasks for routine passaging and on 18–22 mm square coverslips in 6-well plates for co-culture assays. All growth surfaces were coated with growth factor-reduced, phenol red-free Matrigel (Corning) diluted 1:30 in phenol red-free DMEM/F-12 (Gibco, Thermo Fisher Scientific). Matrigel-coated plates were stored at 4 °C and used within 1 week. iPSCs were cultured in mTeSR Plus medium (STEMCELL Technologies) supplemented with 1% penicillin–streptomycin (Cytiva), and medium was replaced daily. Cells were passaged every 3 days at a 1:4–1:8 ratio as single cells by dissociation with Accutase (Gibco, Thermo Fisher Scientific), quenching in 1x Dulbecco’s phosphate-buffered saline (DPBS; Gibco, Thermo Fisher Scientific), and replating onto freshly coated flasks. Media were supplemented with 10 μM Y-27632 ROCK inhibitor (MedChemExpress) for approximately 24 h after passaging and subsequently replaced with ROCK inhibitor-free medium.

### METHOD DETAILS

#### Cell culture and reagents

The following reagents were used at the indicated concentrations: 1 μg mL^−1^ doxycycline (DOX; Millipore-Sigma), 500 μM indole-3-acetic acid (IAA; Millipore-Sigma), 1 μM 5-Ph-IAA (Fisher Scientific), 50 nM CENP-E inhibitor (GSK-923295, Cayman Chemical), 480 nM Mps1 inhibitor (reversine, Fisher Scientific), 100 ng mL^−1^ colcemid (KaryoMAX, Thermo Fisher), 100 ng mL^−1^ nocodazole (Sigma-Aldrich), 400 μg mL^−1^ Geneticin (G418 sulfate, InvivoGen), 2.5 μg mL^−1^ puromycin (Sigma-Aldrich), and 50 μg mL^−1^ zeocin (InvivoGen). For HaloTag-labeling experiments, cells were pre-labeled with 0.1 μM Janelia Fluor 646 HaloTag ligand (Promega) for 3 h and washed with culture medium prior to imaging. For ionizing radiation experiments, cells were irradiated with 2 Gy γ-rays using a Mark I ^137^Cs irradiator (JL Shepherd). siRNA transfections were performed using Lipofectamine RNAiMAX (Thermo Fisher). MAD2L1 siRNAs (SASI_Hs01_00042213; Millipore-Sigma) were used at a final concentration of 20 nM. Cas9 (IDT) ribonucleoproteins were delivered using Lipofectamine CRISPRMAX Cas9 Transfection Reagent (Thermo Fisher) according to the manufacturer’s protocols. The following guide RNA sequences were used: *CENPA* – CCGCGGCCGGTTACCTAAGG; chromosome 3p – ATTACCTTGGGCCCAACATA; *DYZ1* – TGGAATGGAACAGAGAGCAA.

#### Cell line engineering

H2BGFP- or H2B-mCherry-labeled cells were generated by lentiviral infection using PGK-H2BeGFP (Addgene #21210) or PGK-H2BmCherry (Addgene #21217), respectively. H2B-GFP and H2B-mCherry cells were further generated by lentiviral infection with a plasmid expressing CAAX-Halo (a gift from Henry de Belly). dCas9-SunTag HeLa and RPE-1 cells were generated by lentiviral infection with pHRdSV40-dCas9-10xGCN4_v4-P2A-BFP (Addgene #60903) and lentiGuide-scFv-GCN4-sfGFP-GB1-NLS, with sgRNAs targeting Y chromosome DYZ1 repeats, as previously described.^45^ HeLa cells expressing mCherry and zeoR and DLD-1 cells expressing mNeonGreen were generated by retroviral infection with pBABE vectors containing the indicated fluorescent and resistance markers. Viral supernatants were produced in HEK293GP cells co-transfected with pVSV-G using X-tremeGENE 9 (Roche), filtered (0.45 μm), and used to infect target cells in the presence of 5 μg mL^−1^ polybrene (Millipore-Sigma). Infected cells were sorted by FACS.

To engineer RPTECs to induce Y chromosome-specific micronuclei, *CENPA* cDNA was amplified by PCR, cloned into pMK411 (Addgene #140659) to generate OsTIR1(F74G)-P2A-mAID-EGFP-CENP-A, and subcloned into lenti dCAS-VP64_Blast (Addgene #61425). RPTECs were transduced and sorted by FACS (BD FACSAria II) for GFP-positive cells. Expression and auxin-mediated degradation of mAID-EGFP-CENP-A was confirmed by immunoblotting and microscopy. The endogenous *CENPA* genes were then deleted using sgRNAs targeting the intron–exon boundary of exon 1. Ribonucleoprotein complexes containing synthetic guide RNAs (Genscript) and Alt-R S.p. Cas9 Protein V3 (IDT) were assembled and co-transfected using Lipofectamine CRISPRMAX Cas9 Transfection Reagent (Invitrogen). Cells were plated for clonal expansion and clones were screened by immunoblot. To express mutant CENP-A containing the carboxy-terminal tail of histone H3, mutant *CENPA* cDNA was cloned into pCW-Cas9 (Addgene #50661). RPTECs were transduced and selected with puromycin for 7 days.

#### Fluorescence-activated cell sorting

Cells were harvested by trypsinization, washed twice with phosphate-buffered saline (PBS), and resuspended at a concentration of 2 × 10^7^ cells/mL^−1^ in sorting buffer (PBS supplemented with 1% FBS). Cell suspensions were passed through mesh-capped polypropylene tubes (Fisher Scientific and Avantor) prior to sorting. Single-cell suspensions were sorted on a BD FACSAria II cell sorter (BD Biosciences) with a 100 μM nozzle and equipped with FACSDIVA software (v8.0.2). Data were analyzed using FlowJo software (v10). Forward- and side-scatter parameters were used to exclude debris, and doublets were excluded using FSC-A versus FSC-W gating. For the experiments described in Figure 4, Gates for each fluorescent population (mCherry+, mNeonGreen+, and double positive cells) were established using single-fluorescent parental cell lines and compared to the corresponding non-fluorescent parental control lines (as shown in Figure S5A).

#### Live-cell imaging

Live-cell imaging was performed on unlabeled and H2B-labeled RPE-1 cells in 35 mm glass-bottom dishes (MatTek) on a DeltaVision Ultra microscope (GE Healthcare) in a 37 °C, 5% CO_2_-controlled chamber. Images were acquired at 5-min intervals over 16 h using a 20x objective and 7 × 0.5 μm z-sections under low-power exposure. To visualize DNA, cells were pre-labeled with 1 μM SiR-DNA (Cytoskeleton Inc.) for 2 h. CENP-E and Mps1 inhibitors (50 nM and 480 nM, respectively), along with cytochalasin D (5 μM, a gift from Courtney Schroeder), were added immediately prior to imaging. Deconvolution and maximum-intensity projections were performed using softWoRx (v7.2.1, Cytiva), and images were analyzed using Fiji (v2.1.0/1.53c).

Live-cell imaging was performed on H2B-mCherry-labeled RPTECs in 35 mm glass-bottom dishes (MatTek) on a CellVoyager CQ1 Benchtop High-Content Analysis System (Yokogawa) in a 37 °C, 5% CO_2_-controlled chamber. Images were acquired at 10-min intervals over 48 h using a 40x objective and 11 × 0.5 μm z-sections under low-power exposure. Maximum-intensity projections were performed using CQ1 software (v1.08.02.01, Yokogawa), and images were analyzed using Fiji (v2.1.0/1.53c).

#### Immunofluorescence

For immunostaining of nanotubes, cells were fixed in 100% methanol for 3 min, followed by a 3-min wash in TBS (0.15 M NaCl, 0.02 M Tris-HCl, pH 7.4). Samples were permeabilized in TBS containing 0.5% Triton X-100 for 10 min, then washed three times for 5 min each in TBS–0.1% Triton X-100. Blocking was performed for 10 min in freshly prepared blocking solution (TBS–0.1% Triton X-100, 2% BSA, 0.1% sodium azide). Samples were incubated for 2 h at room temperature with primary antibodies diluted in blocking solution: anti-β-actin (1:100, #4970S, Cell Signaling), anti-α-tubulin (1:500, #3873, Cell Signaling; 1:250, MA1–80017, Invitrogen), and anti-CENP-A (1:500, ADI-KAM-CC006-E, Enzo). After five 30-min washes with TBS–0.1% Triton X-100, Alexa Fluor-conjugated donkey anti-rabbit, -mouse, or -rat secondary antibodies (1:1,000, Invitrogen) were applied for 2 h at room temperature. After one 5-min wash in TBS–0.1% Triton X-100 and one rinse in TBS, DNA was counterstained with YOYO-1 (1 μM, Invitrogen), and samples were mounted in ProLong Gold Antifade Mountant. Imaging was performed on a DeltaVision Ultra microscope and analyzed using Fiji (v2.1.0/1.53c).

To label the cell membrane, RPE-1 cells were plated onto chambered glass slides for 16 h. Cells were fixed in 4% paraformaldehyde (PFA) for 10 min at room temperature, washed 3x in PBS, then treated with cold methanol for 5 min on ice. Following 3x PBS washes, samples were blocked in 0.2 M glycine, 2.5% FBS, and 0.1% Triton X-100 in PBS for 1 h at room temperature. Cells were incubated overnight at 4 °C with anti-ZO-1 (1:1,000, #AB2272, Sigma-Aldrich), washed 3x in PBS, then incubated with Alexa Fluor-conjugated donkey anti-rabbit secondary antibody (1:1,000, Invitrogen) for 1 h. After washing, samples were counterstained with DAPI, air-dried, and mounted in ProLong Gold. Imaging was performed on a DeltaVision Ultra microscope and analyzed using Fiji (v2.1.0/1.53c).

#### DNA transfer in human iPSCs

The following methods describe experiments specific to human iPSCs. For co-culture assays, Lamin B1-RFP and α-tubulin-GFP iPSCs were co-cultured at a 1:1 ratio and seeded onto Matrigel-coated coverslips. Co-cultures were maintained for 72 h under standard iPSC culture conditions. Cells were rinsed in 1x PHEM buffer and fixed in 4% PFA prepared in 1x PHEM for 10 min at room temperature, followed by three washes in 1x PBS. DNA was visualized by pre-labeling live cells through incubation in mTeSR Plus medium containing 100 nM SPY650 DNA dye (Cytoskeleton) for 1 h at 37 °C prior to fixation, followed by washing in 1x PBS. Coverslips were mounted in Vectashield HardSet mounting medium (Vector Laboratories). Imaging was performed on a Zeiss Axioplan II epifluorescence microscope at room temperature using a Hamamatsu ORCA II camera and a 100x/1.4 NA oil-immersion objective. Images were acquired using MetaMorph (RRID: SCR_002368). Z-stacks were deconvolved in MetaMorph using the nearest-neighbors algorithm and processed in Fiji (RRID: SCR_002285).

#### Labeling with thymidine analogs

RPE-1 cells were labeled with IdU or CldU (25 μM, Sigma-Aldrich) for 72 h, with CENP-E/Mps1 inhibitors added during the final 24 h. Cells were trypsinized, mixed 1:1, and co-cultured for 24 h. Cells were treated with colcemid (100 ng mL^−1^, KaryoMAX, Thermo Fisher) for 4 h, and mitotic cells were collected by shake-off and incubated in 75 mM KCl for 5 min at 37 °C. Mitotic chromosome spreads were prepared using a Cytospin 4 (Thermo Fisher), fixed with 4% PFA for 10 min, and washed 3x in PBS. Samples were post-fixed with 2.5 M HCl, rinsed twice in water, and blocked in 3% BSA in PBS for 1 h. Primary antibodies against BrdU were applied for 1 h: 1:250 anti-BrdU (clone B44, #347580, BD Biosciences) and 1:500 anti-BrdU [BU1/75 (ICR1), #AB6326, Abcam]. After 3x PBS washes, samples were fixed again in 4% PFA, washed, and incubated with Alexa Fluor-conjugated donkey anti-rat or anti-mouse secondary antibodies (1:1,000, Invitrogen) for 2 h. Samples were washed, DAPI-stained, and mounted in ProLong Gold. Imaging was performed on a DeltaVision Ultra microscope and analyzed using Fiji (v2.1.0/1.53c).

#### Mitotic chromosome spreads

For immunofluorescence, cells were treated with colcemid (100 ng mL^−1^, KaryoMAX, Thermo Fisher) for 4 h, and mitotic cells were collected by shake-off and incubated in 75 mM KCl for 5 min at 37 °C. Mitotic chromosome spreads were prepared using a Cytospin 4 (Thermo Fisher), fixed with 4% PFA for 10 min, washed 3x in PBS, DAPI-stained, and mounted in ProLong Gold. Imaging was performed on a DeltaVision Ultra microscope and analyzed using Fiji (v2.1.0/1.53c).

For DNA FISH, cells were treated with colcemid (100 ng mL^−1^) for 4 h, trypsinized, and pelleted by centrifugation. Pellets were resuspended in 500 μl PBS, followed by dropwise addition of 5 mL 75 mM KCl while vortexing gently. Cells were incubated at 37 °C for 6 min, fixed in cold Carnoy’s fixative (3:1 methanol:acetic acid), centrifuged, and resuspended in fresh fixative. Metaphase spreads were dropped onto slides, air-dried, stained with DAPI, rinsed in PBS, and mounted in ProLong Gold.

#### DNA fluorescence in situ hybridization

For multiplex FISH, 24XCyte human multicolor FISH probes (MetaSystems) were used. For locus-specific FISH, probes targeting the Yq11.221 locus were derived from bacterial artificial chromosomes (RP11–113K10, BACPAC Resources) and labeled with SEEBRIGHT orange 552 dUTP by nick translation (Enzo). RP11–113K10 BAC probes were diluted 1:1 with Y chromosome-specific paint probes (MetaSystems), denatured at 75 °C for 5 min, and applied to slides with metaphase spreads. Slides were sealed with coverslips using rubber cement and co-denatured at 75 °C for 2 min. Hybridization was performed overnight at 37 °C in a humidified chamber. Slides were washed in 0.4x SSC at 72 °C for 2 min and rinsed in 2x SSC/0.05% Tween-20 at room temperature. Slides were counterstained with DAPI, air-dried, and mounted in ProLong Gold. Images were acquired using the Metafer Scanning and Imaging Platform (Metafer 4, v3.13.6, MetaSystems). Metaphase identification and capture were automated using 10x and 63x objectives, respectively, and images were analyzed with Isis (MetaSystems) and Fiji (v2.1.0/1.53c).

#### SABER-FISH

Primer exchange reactions (PER) were performed to extend FISH probes into concatemerized SABER probes. PER reactions contained 1x PBS, 10 mM MgSO_4_, 300 μM dNTP mix (dA, dC, and dT only), 100 nM Clean.G hairpin, 600 U mL^−1^ Bst LF polymerase (New England Biolabs), 1 μM hairpin, and nuclease-free water to a final volume of 90 μL. Reactions were incubated at 37 °C for 15 min, after which 10 μL of 10 μM unextended probe was added. Reactions were incubated at 37 °C for 4 h, followed by heat inactivation at 80 °C for 20 min. To assess concatemer length, samples were diluted in 2x Novex TBE–Urea Sample Buffer, boiled at 95 °C for 5 min, and resolved on a 10% TBE–urea gel for 40 min at 70 °C using a circulating water bath connected to the gel cassette. Completed reactions were dehydrated by vacuum evaporation at 60 °C and resuspended in hybridization buffer for downstream FISH experiments.

For fluorescent SABER-FISH on metaphase spreads, slides were denatured in 2x SSC/0.1% Tween-20 containing 70% formamide at 80 °C for 3 min in a water bath, followed by sequential dehydration in ice-cold 70%, 90%, and 100% ethanol for 5 min each. Slides were air-dried and incubated with 25 μL primary hybridization solution consisting of 2x SSC, 0.1% Tween-20, 50% formamide, 10% dextran sulfate, 0.4 μg μL^−1^ RNase A, and 30 pmol unpurified PER product. Slides were cover-slipped, sealed with rubber cement, and hybridized overnight at 37 °C in a humidified chamber. The following day, coverslips were removed and slides were washed four times in 2x SSC/0.1% Tween-20 at 60 °C for 5 min each, followed by two washes in 2x SSC/0.1% Tween-20 at room temperature and one wash in 1x PBS. Secondary hybridization was performed by applying 150 μL secondary hybridization buffer containing 0.2–0.4 μM fluorescent oligonucleotides in 1x PBS. Slides were cover-slipped and incubated at 37 °C for 1 h, then washed twice in 1x PBS/0.1% Tween-20 at 37 °C and once in 1x PBS for 5 min each. Slides were counterstained with 1 μg mL^−1^ DAPI for 5 min, mounted in SlowFade Antifade Mountant, cover-slipped, and sealed with clear nail polish.

Confocal imaging of SABER-FISH samples were performed using a Yokogawa CSU-W1 SoRa spinning disk confocal system mounted on a Nikon Eclipse Ti2 microscope. Excitation was provided by 405-nm, 561-nm, or 640-nm lasers operated at 30% of maximal output from a Nikon LUNF 405/488/561/640 nm laser launch. Laser excitation was delivered via a single-mode optical fiber into the CSU-W1 SoRa unit, passed through a microlens array disk and a SoRa disk containing 50-μm pinholes, and directed to the rear aperture of a 100x/1.49 NA Apo TIRF oil-immersion objective lens. Emission light was collected through the same objective, relayed back through the SoRa unit, and directed into the emission path by a quadband dichroic mirror (Semrock Di01-T405/488/568/647–13×15×0.5). Emission signals were filtered using single-bandpass filters (DAPI: Chroma ET455/50M; ATTO 565: Chroma ET605/50M; Alexa Fluor 647: Chroma ET705/72M) and focused onto an Andor Sona 4.2B11 camera (11 μm physical pixel size), yielding an effective pixel size of 110 nm. Images were acquired in 16-bit mode with rolling shutter readout and 300 ms exposure times using Nikon Elements AR v5.20.00.

#### Immunoblotting

Whole-cell lysates were collected in Laemmli SDS sample buffer and boiled for 10 min. Proteins were separated by SDS-PAGE, transferred to PVDF membranes, and blocked in 5% milk in PBST (PBS with 0.1% Tween-20). Membranes were probed with anti-CENP-A (1:1,000, #2186, Cell Signaling), anti-neomycin phosphotransferase II (1:1,000, #06–747, Sigma-Aldrich), anti-UTY (1:1,000, #48779S, Cell Signaling), and anti-α-tubulin (1:5,000, #3873, Cell Signaling), followed by incubation with HRP-conjugated secondary antibodies (1:10,000, Sigma-Aldrich). Signal was detected using SuperSignal West Pico Plus substrate (Thermo Fisher) and imaged on a ChemiDoc MP system (Bio-Rad).

#### Reverse transcription PCR

Total RNA was extracted using a RNeasy Mini Kit (Qiagen). RNA concentration and purity were assessed by A260/A280 ratio on a NanoDrop (Thermo Fisher). First-strand complementary DNA (cDNA) was synthesized using an iScript Reverse Transcription Supermix kit (Bio-Rad) following the manufacturer’s protocol. PCR amplifications were performed using Q5 PCR master mix (NEB) with the indicated primers: neoR forward (GATCTCCTGTCATCTCACCTTG), neoR reverse (GCCAACGCTATGTCCTGATA); *UTY* forward (TCACCCTCTTCAGCCATTTC), *UTY* reverse (GGTCTTGGAGGTGGACATTTAT); *GAPDH* forward (ACATCGCTCAGACACCATGG), *GAPDH* reverse (GTAGTTGAGGTCAATGAAGGG). PCR products were resolved by electrophoresis on a 2% agarose gel containing ethidium bromide and visualized under UV illumination.

#### Single-cell RNA sequencing

Single-cell RNA sequencing (scRNA-seq) libraries were prepared by the UT Southwestern McDermott Center Sequencing Core using the 10x Genomics Chromium X platform. Cells were processed with the GEM-X Single Cell 3′ Reagent Kit v4, combined with GEM-X Single Cell 3′ Gel Beads v4, and loaded onto a GEM-X 3′ Chip. Libraries were generated from gel bead-in-emulsions (GEMs), quality-controlled by Tapestation and Qubit, and sequenced at a 2 nM concentration on an Illumina NovaSeq X 10B flow cell.

Raw sequencing reads were aligned to the GRCh38 build of the human reference genome using Cell Ranger v.10.0.0 and default parameter values. Prior to alignment, we extended the GRCh38 build of the human reference genome by including the lentiviral vector sequences of mCherry, mNeonGreen and neoR as additional contigs using the *mkref*() function from the Cell Ranger software.^79^ Cells with fewer than 4,500 detected genes or with mitochondrial gene expression exceeding 5% were excluded. In addition, genes expressed in fewer than three cells were removed. Count matrices were normalized using the *NormalizeData*() function from the R package Seurat^80^ using the normalization method “LogNormalize”. Differentially expressed genes between parental donor and recipient cells were identified using the *FindAllMarkers*() function from Seurat. We constructed a nearest-neighbor graph for the mCherry+ data set using the *FindNeighbors*() function from Seurat. Next, we performed unsupervised clustering using the Louvain algorithm^81^ as implemented in the *FindClusters*() function from Seurat using a resolution of 0.1, which identified 4 cell clusters. Finally, we performed non-linear dimensionality reduction through Uniform Manifold Approximation and Projection using the *RunUMAP*() function from Seurat. Gene expression was visualized using the *DotPlot*() and *FeaturePlot*() functions from Seurat.

### QUANTIFICATION AND STATISTICAL ANALYSIS

Statistical analyses were performed as described in the figure legends using GraphPad Prism v9.5.0. Sample sizes, statistical tests, and significance values are indicated in the main text, figure, or figure legends. *P* ≤ 0.05 was considered statistically significant. Error bars represent standard error mean (SEM) unless indicated otherwise.

## Supplementary Material

1

2

3

4

5

6

7

8

9

10

11

12

13

14

Supplemental information can be found online at https://doi.org/10.1016/j.cell.2026.04.041.

## Figures and Tables

**Table:** KEY RESOURCES TABLE

REAGENT or RESOURCE	SOURCE	IDENTIFIER
Antibodies		
Rabbit monoclonal anti-β-actin antibody	Cell Signaling	Cat# 4970
Mouse monoclonal anti-α-tubulin antibody	Cell Signaling	Cat# 3873
Rat monoclonal anti-α-tubulin antibody	Invitrogen	Cat# MA1-80017
Mouse monoclonal anti-CENP-A antibody	Enzo	Cat# ADI-KAM-CC006-E
Rabbit polyclonal anti-ZO-1 antibody	Sigma-Aldrich	Cat# AB2272
Mouse monoclonal anti-BrdU clone B44 antibody	BD Biosciences	Cat# #347580
Rat monoclonal Anti-BrdU BU1/75 (ICR1) antibody	Abcam	Cat# AB6326
Rabbit polyclonal anti-CENP-A antibody	Cell Signaling	Cat# #2186
Rabbit polyclonal anti-neomycin phosphotransferase II antibody	Sigma-Aldrich	Cat# #06-747
Rabbit monoclonal anti-UTY antibody	Cell Signaling	Cat# 48779S
Donkey anti-Mouse IgG (H+L) Highly Cross-Adsorbed Secondary Antibody, Alexa Fluor 350	Invitrogen	Cat# A10035
Donkey anti-Mouse IgG (H+L) Highly Cross-Adsorbed Secondary Antibody, Alexa Fluor 488	Invitrogen	Cat# A-21202
Donkey anti-Mouse IgG (H+L) Highly Cross-Adsorbed Secondary Antibody, Alexa Fluor 555	Invitrogen	Cat# A-31570
Donkey anti-Mouse IgG (H+L) Highly Cross-Adsorbed Secondary Antibody, Alexa Fluor 647	Invitrogen	Cat# A-31571
Donkey anti-Rabbit IgG (H+L) Highly Cross-Adsorbed Secondary Antibody, Alexa Fluor 350	Fisher Scientific	Cat# A10039
Donkey anti-Rabbit IgG (H+L) Highly Cross-Adsorbed Secondary Antibody, Alexa Fluor 488	Invitrogen	Cat# A-21206
Donkey anti-Rabbit IgG (H+L) Highly Cross-Adsorbed Secondary Antibody, Alexa Fluor 555	Invitrogen	Cat# A-31572
Donkey anti-Rabbit IgG (H+L) Highly Cross-Adsorbed Secondary Antibody, Alexa Fluor 647	Invitrogen	Cat# A-31573
Donkey anti-Rat IgG (H+L) Highly Cross-Adsorbed Secondary Antibody, CF 405S	Biotium	Cat# 20419
Donkey anti-Rat IgG (H+L) Highly Cross-Adsorbed Secondary Antibody, Alexa Fluor 488	Invitrogen	Cat# A-21208
Donkey Anti-Mouse IgG Antibody, HRP conjugate, Species Adsorbed	Sigma-Aldrich	Cat# AP192P
Goat Anti-Rabbit IgG Antibody, HRP conjugate, Species Adsorbed	Sigma-Aldrich	Cat# AP187P
Bacterial and virus strains		
NEB 5-alpha competent cells	New England Biolabs	Cat# C2987U
Chemicals, peptides, and recombinant proteins		
High-glucose DMEM	Gibco	Cat# 11965092
DMEM/F-12	Gibco	Cat# 11320033
Tetracycline-free fetal bovine serum (FBS)	Omega Scientific	Cat# FB-16
REGM Renal Epithelial Cell Growth Medium BulletKit	Lonza	Cat# CC-3190
mTeSR Plus medium	STEMCELL Technologies	Cat# 100-1130
Penicillin-streptomycin	Sigma-Aldrich	Cat# P4333
Penicillin-streptomycin	Cytiva	Cat# SV30010
Doxycycline	Sigma-Aldrich	Cat# D3447
Indole-3-acetic acid sodium salt (auxin)	Sigma-Aldrich	Cat# I5148
5-Ph-IAA	Fisher Scientific	Cat# 50-225-8953
CENP-E inhibitor GSK-923295	Cayman Chemical	Cat# 1088965-37-0
Mps1 inhibitor reversine	Fisher Scientific	Cat# 50-289-0846
Colcemid (KaryoMAX)	Gibco	Cat# 15212012
Nocodazole	Millipore-Sigma	Cat# 487928
Geneticin (G418 Sulfate)	Invivogen	Cat# Ant-gn-5
Puromycin	Sigma-Aldrich	Cat# P8833
Zeocin	InvivoGen	Cat# Ant-zn-05
SPY650 DNA dye	Cytoskeleton	Cat# CY-SC501
Vectashield HardSet mounting medium	Vector Laboratories	Cat# H-1700
Matrigel Growth Factor Reduced (GFR) Basement Membrane Matrix	Corning	Cat# CLS356231
StemPro Accutase Cell Dissociation Reagent	Thermo Fisher Scientific	Cat# A1110501
Y-27632 ROCK inhibitor	MedChemExpress	Cat# HY-10583
5-Iodo-2′-deoxyuridine (IdU)	Sigma-Aldrich	Cat# I7125
5-Chloro-2′-deoxyuridine (CldU)	Sigma-Aldrich	Cat# C6891
SuperSignal West Pico Plus	Thermo Fisher Scientific	Cat# 34580
ProLong Gold Antifade Mountant	Invitrogen	Cat# P36930
Janelia Fluor 646 HaloTag ligand	Promega	Cat# GA1120
Lipofectamine RNAiMAX	Thermo Fisher Scientific	Cat# 13778030
Lipofectamine^™^ CRISPRMAX^™^ Cas9	Thermo Fisher Scientific	Cat# CMAX00008
X-tremeGENE 9	Roche	Cat# 6365779001
Polybrene	Sigma-Aldrich	Cat# TR-1003
Alt-R S.p. Cas9 Protein V3	IDT	Cat# 1081058
SiR-DNA	Cytoskeleton	Cat# CY-SC007
Cytochalasin D	Fisher Scientific	Cat# PHZ1063
YOYO-1	Invitrogen	Cat# Y3601
Critical commercial assays		
Nick Translation DNA Labeling System 2.0	Enzo Life Sciences	Cat# ENZ-GEN111-0050
RNeasy Mini Kit	Qiagen	Cat# 74104
iScript Reverse Transcription Supermix kit	Bio-Rad	Cat# 1708841
GEM-X Single Cell 3′ Reagent Kit v4	10x Genomics	Cat# PN-1000691
Universal Mycoplasma Detection Kit	ATCC	Cat# 30-1012K
Deposited data		
Single-cell RNA sequencing data	This study	ENA: PRJEB107007
Experimental models: Cell lines		
DLD-1 CEN-SELECT + mNeonGreen	This study	N/A
HeLa S3 + mCherry and zeoR	This study	N/A
HeLa S3 + H2B-mCherry	This study	N/A
HeLa S3 + H2B-GFP	This study	N/A
RPE-1	ATCC	CRL-4000
RPE-1 + dnTRF2	John Maciejowski	Maciejowski et al.^57^
RPE-1 + H2B-mCherry	This study	N/A
RPE-1 + H2B-GFP	This study	N/A
RPE-1 + H2B-mCherry + CAAX-Halo	This study	N/A
RPE-1 + H2B-GFP + CAAX-Halo	This study	N/A
RPE-1 + dCas9-SunTag	This study	N/A
RPTEC	ATCC	CRL-4031
RPTEC CENPA−/− + mAID-GFP-CENP-A	This study	N/A
RPTEC CENPA−/− + mAID-GFP-CENP-A + CENP-A mutant	This study	N/A
RPTEC + H2B-mCherry	This study	N/A
RPTEC + H2B-GFP	This Study	N/A
iPSC: WTC-mTagRFPT-LMNB1-cl62 (LMNB1-mTagRFP-T)	Coriell Institute for Medical Research	Cat# AICS-0034-062; RRID: CVCL_ZX21
iPSC: WTC-mEGFP-TUBA1B-cl105 (TUBA1B-mEGFP)	Coriell Institute for Medical Research	Cat# AICS-0012; RRID: CVCL_IR34
293T	ATCC	CRL-3216
293GP	Burns et al.^82^	N/A
Oligonucleotides		
neoR forward primer: GATCTCCTGTCATCTCACCTTG	This study	N/A
neoR reverse primer: GCCAACGCTATGTCCTGATA	This study	N/A
*UTY* forward primer: TCACCCTCTTCAGCCATTTC	Ly et al.^43^	N/A
*UTY* reverse primer: GGTCTTGGAGGTGGACATTTAT	Ly et al.^43^	N/A
*GAPDH* forward primer: ACATCGCTCAGACACCATGG	Ly et al.^43^	N/A
*GAPDH* reverse primer: GTAGTTGAGGTCAATGAAGGG	Ly et al.^43^	N/A
MAD2L1 siRNA	Millipore Sigma	SASI_Hs01_00042213
CENPA gRNA: CCGCGGCCGGTTACCTAAGG	This study	N/A
Chromosome 3p gRNA: ATTACCTTGGGCCCAACATA	This study	N/A
DYZ1 gRNA: TGGAATGGAACAGAGAGCAA	Lin et al.^45^	N/A
Recombinant DNA		
PGK-H2BeGFP	Kita-Matsuo et al.^83^	Addgene #21210
PGK-H2BmCherry	Kita-Matsuo et al.^83^	Addgene #21217
pHR-SFFV-NES-Halo-CAAX	De Belly et al.^84^	N/A
pHRdSV40-dCas9-10xGCN4_v4-P2A-BFP	Tanenbaum et al.^60^	Addgene #60903
lentiGuide-scFv-GCN4-sfGFP-GB1-NLS	Lin et al.^45^	N/A
pBABE-mCherry-zeoR	This study	N/A
pBABE-mNeonGreen	This study	N/A
pMK411 (OsTIR1(F74G) mAID-EGFP-Nluc)	Yesbolatova et al.^85^	Addgene #140659
pCW-Cas9	Wang et al.^86^	Addgene #50661
dCAS-VP64_Blast	Konermann et al.^87^	Addgene #61425
Software and algorithms		
Fiji v2.1.0/1.53c	Schindelin et al.^88^	N/A
GraphPad Prism v9.5.0	GraphPad Software Inc.	N/A
Adobe Creative Cloud	Adobe Inc.	N/A
Metafer 4 v3.13.6	MetaSystems	N/A
Isis	MetaSystems	N/A
softWorX v.7.2.1	Cytiva	N/A
FlowJo software (v10)	FlowJo	N/A
CQ1 software v1.08.02.01	Yokogawa	N/A
MetaMorph Microscopy Automation and Image Analysis Software version 7.8.0.0	Molecular Devices	RRID:SCR_002368
Other		
XCP Y Green DNA FISH probe	MetaSystems	Cat# D-0324-050-FI
XCP X Orange DNA FISH probe	MetaSystems	Cat# D-0323-050-OR
Human multicolor DNA FISH probe	MetaSystems	Cat# D-0125-060-DI
Yq11.221 locus BAC probe - RP11-113K10	BACPAC Resources	RP11-113K10

## Highlights

- Genomic instability generates micronuclei and chromosome fragments in the cytoplasm
- Direct cell-cell contact triggers intercellular transfer of genomic DNA
- Transferred DNA fragments are functional and maintained within recipient cell genomes
- Intercellular DNA transfer can confer heritable phenotypic changes
