# Supplementary material for: Genome instability triggers intercellular DNA transfer between human cells
Source: Cell. Author manuscript; Available in PMC 2026 May 22. (PMC13193222; doi:10.1016/j.cell.2026.04.041)
Supplement: 1 [file NIHMS2176727-supplement-1.pdf]

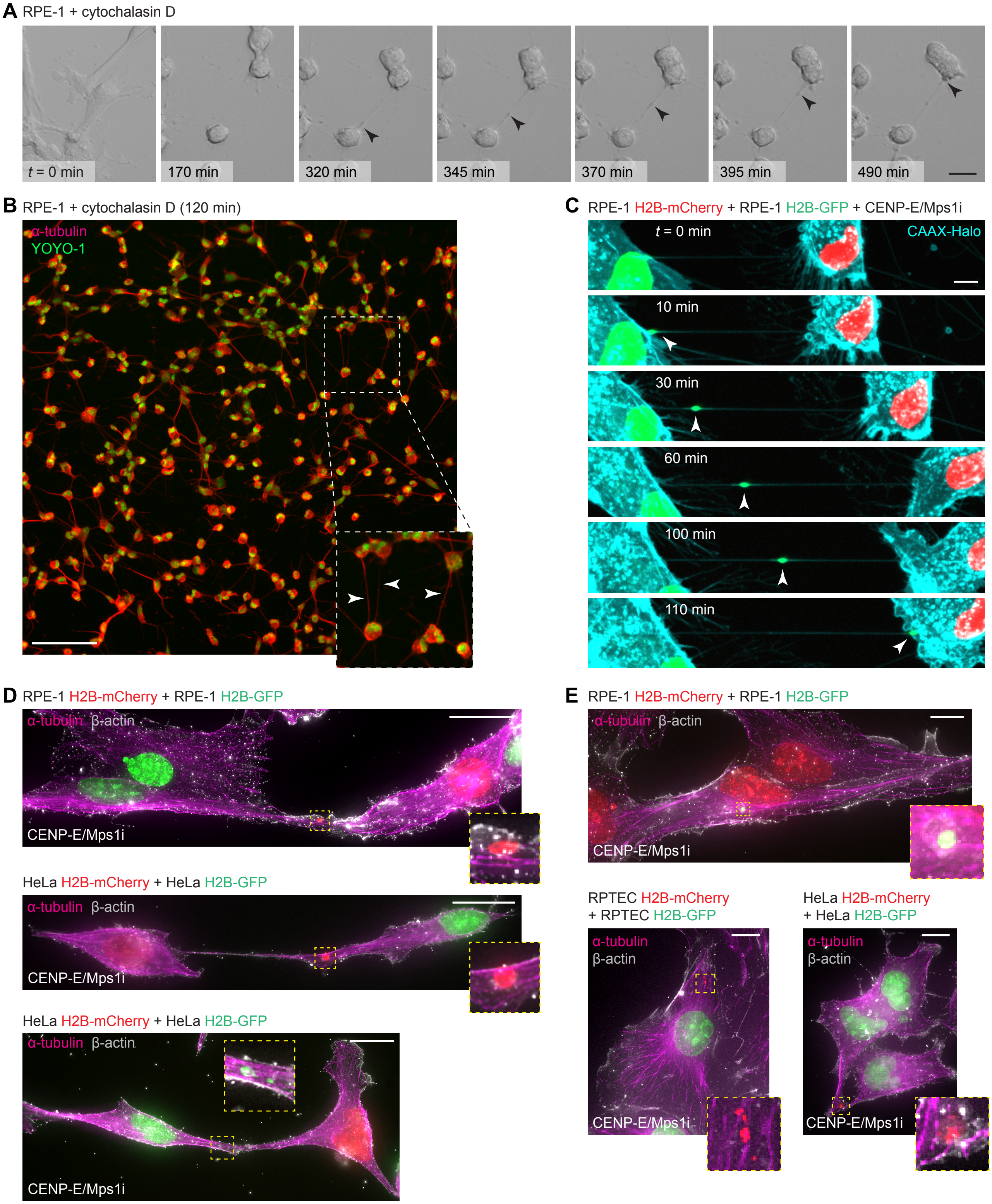
A RPE-1 + cytochalasin D
t = 0 min
170 min
320 min
345 min
370 min
395 min
490 min
B RPE-1 + cytochalasin D (120 min)
α-tubulin
YOYO-1
C RPE-1 H2B-mCherry + RPE-1 H2B-GFP + CENP-E/Mps1i
t = 0 min
CAAX-Halo
10 min
30 min
60 min
100 min
110 min
D RPE-1 H2B-mCherry + RPE-1 H2B-GFP
α-tubulin β-actin
CENP-E/Mps1i
HeLa H2B-mCherry + HeLa H2B-GFP
α-tubulin β-actin
CENP-E/Mps1i
HeLa H2B-mCherry + HeLa H2B-GFP
α-tubulin β-actin
CENP-E/Mps1i
E RPE-1 H2B-mCherry + RPE-1 H2B-GFP
α-tubulin β-actin
CENP-E/Mps1i
RPTEC H2B-mCherry
+ RPTEC H2B-GFP
α-tubulin
β-actin
CENP-E/Mps1i
HeLa H2B-mCherry
+ HeLa H2B-GFP
α-tubulin
β-actin
CENP-E/Mps1i
