# Supplementary figures and images for: Genome instability triggers intercellular DNA transfer between human cells
Source: Cell. Author manuscript; Available in PMC 2026 May 22. (PMC13193222; doi:10.1016/j.cell.2026.04.041)

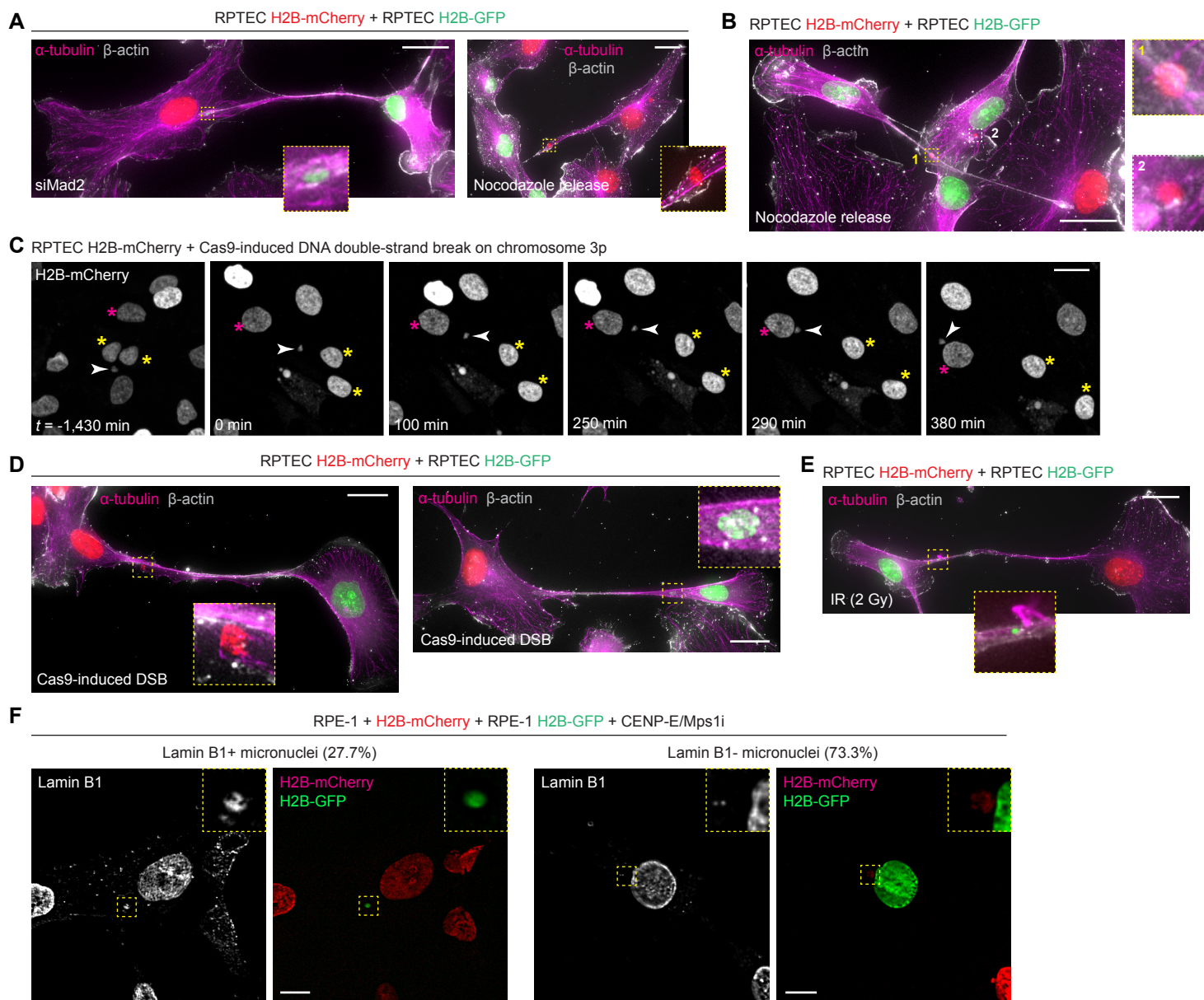

Supplement: 2 [file NIHMS2176727-supplement-2.pdf]

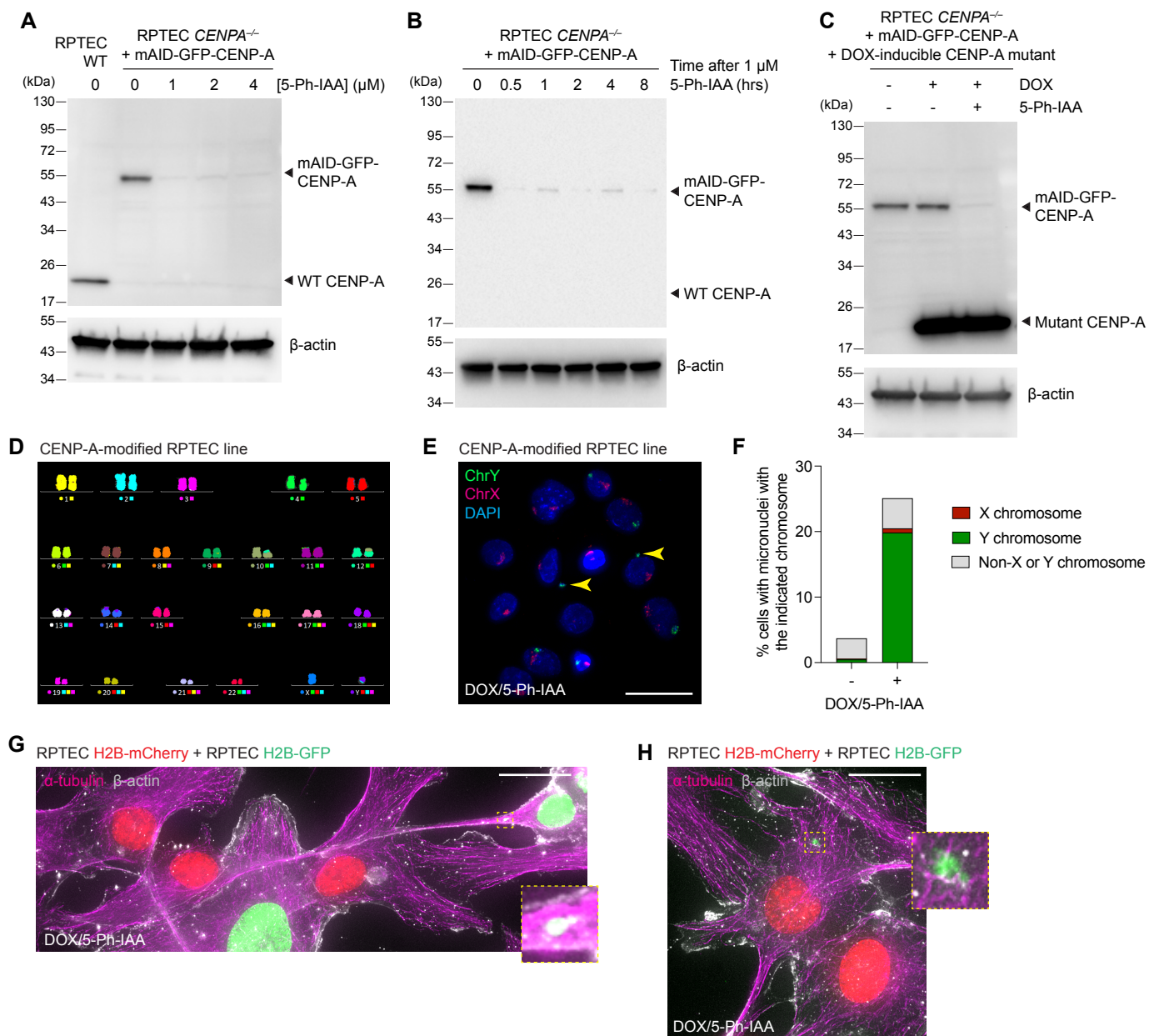

Supplement: 3 [file NIHMS2176727-supplement-3.pdf]

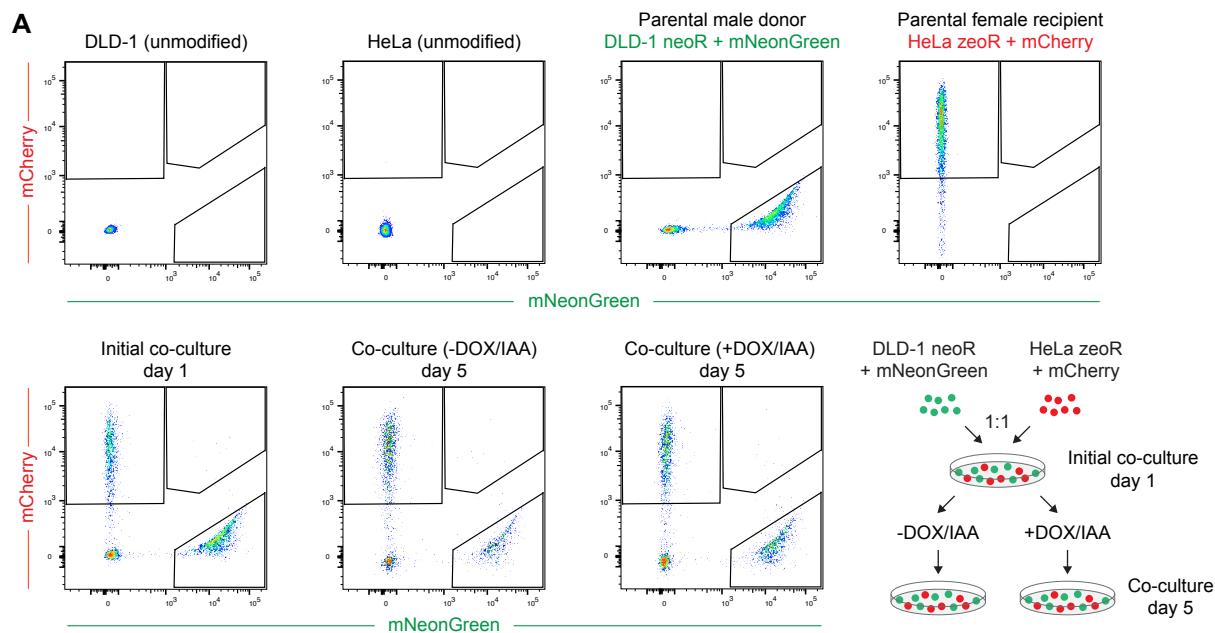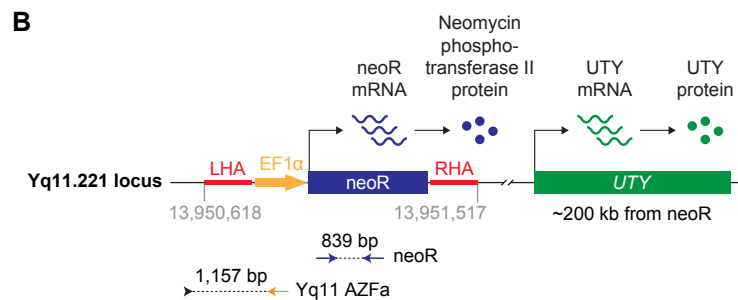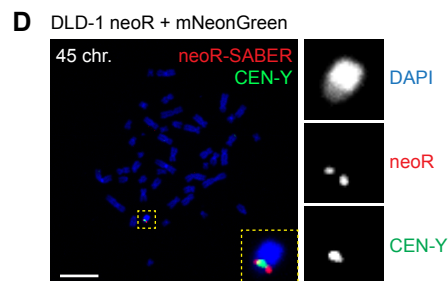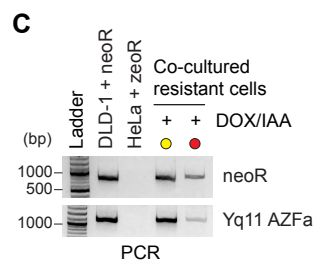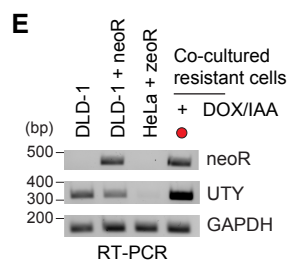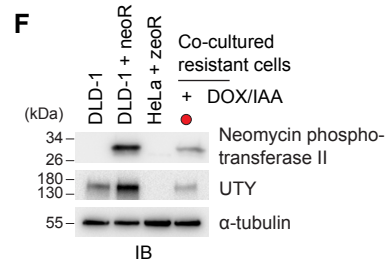

Supplement: 5 [file NIHMS2176727-supplement-5.pdf]

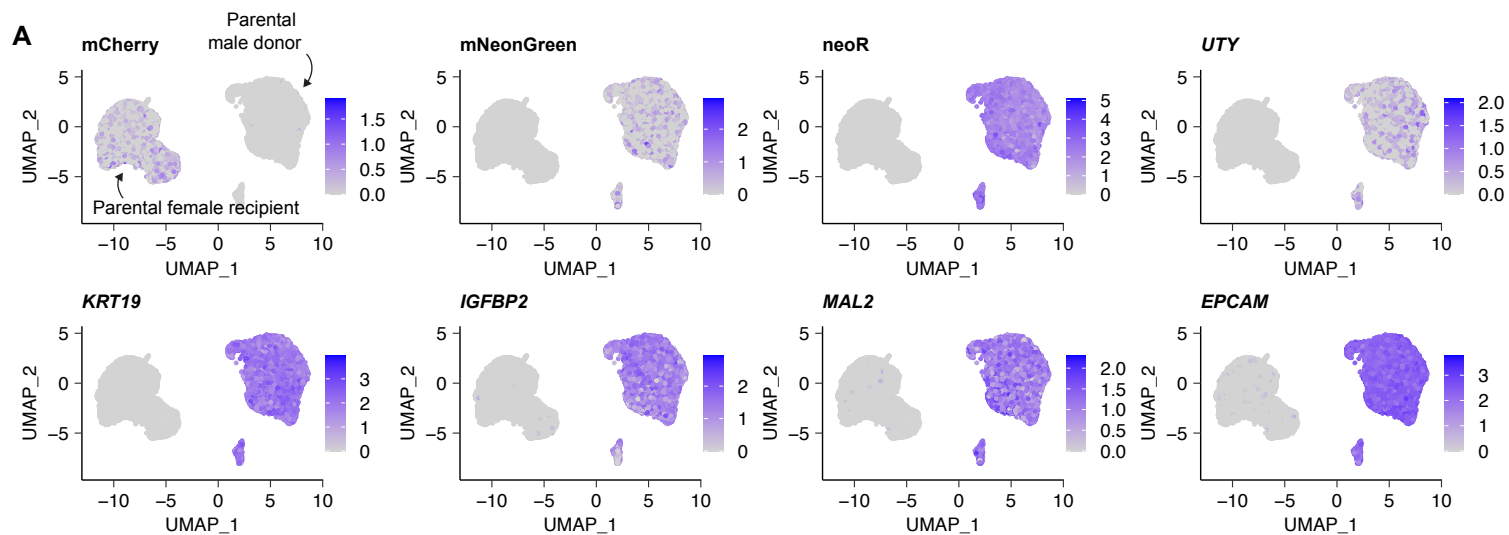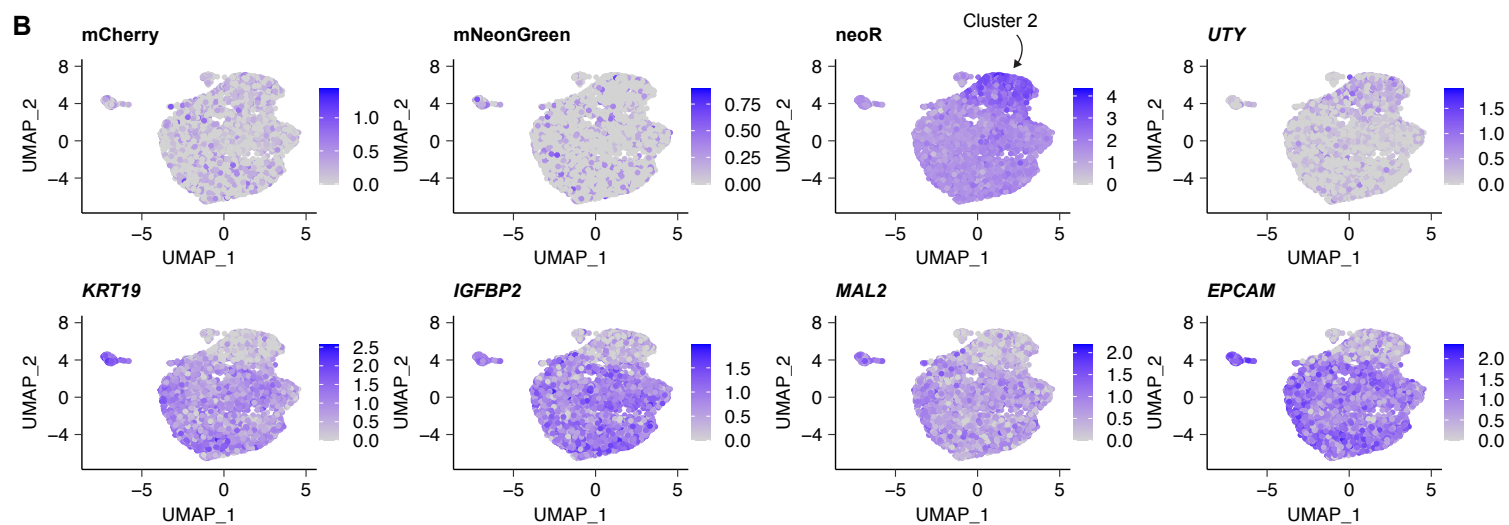

Supplement: 6 [file NIHMS2176727-supplement-6.pdf]
