# Supplementary material for: Genome instability triggers intercellular DNA transfer between human cells
Source: Cell. Author manuscript; Available in PMC 2026 May 22. (PMC13193222; doi:10.1016/j.cell.2026.04.041)
Supplement: 4 [file NIHMS2176727-supplement-4.pdf]

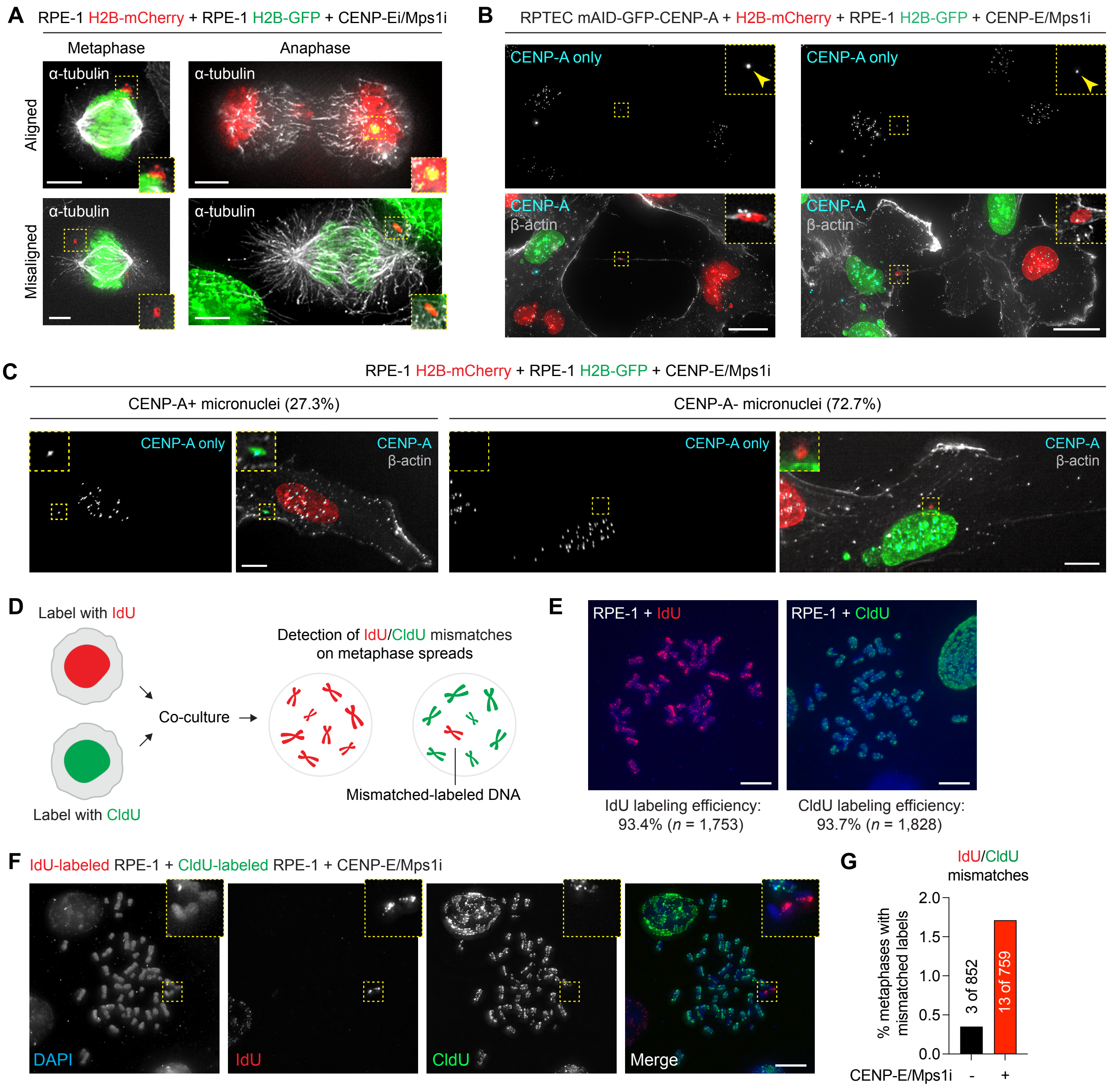

A
RPE-1 H2B-mCherry + RPE-1 H2B-GFP + CENP-Ei/Mps1i
Metaphase
Anaphase
Aligned
Misaligned
α-tubulin
B
RPTEC mAID-GFP-CENP-A + H2B-mCherry + RPE-1 H2B-GFP + CENP-E/Mps1i
CENP-A only
CENP-A
β-actin
C
RPE-1 H2B-mCherry + RPE-1 H2B-GFP + CENP-E/Mps1i
CENP-A+ micronuclei (27.3%)
CENP-A- micronuclei (72.7%)
D
Label with IdU
Label with CldU
Co-culture
Detection of IdU/CldU mismatches on metaphase spreads
Mismatched-labeled DNA
E
RPE-1 + IdU
RPE-1 + CldU
IdU labeling efficiency: 93.4% (n = 1,753)
CldU labeling efficiency: 93.7% (n = 1,828)
F
IdU-labeled RPE-1 + CldU-labeled RPE-1 + CENP-E/Mps1i
DAPI
IdU
CldU
Merge
G
IdU/CldU mismatches
% metaphases with mismatched labels
2.0
1.5
1.0
0.5
0.0
3 of 852
13 of 759
CENP-E/Mps1i
-
+
